# Anticancer Activity of Metallodrugs and Metallizing Host Defense Peptides—Current Developments in Structure-Activity Relationship

**DOI:** 10.3390/ijms25137314

**Published:** 2024-07-03

**Authors:** Celia María Curieses Andrés, José Manuel Pérez de la Lastra, Elena Bustamante Munguira, Celia Andrés Juan, Eduardo Pérez-Lebeña

**Affiliations:** 1 Hospital Clínico Universitario of Valladolid, Avenida de Ramón y Cajal, 3, 47003 Valladolid, Spain; cmcurieses@gmail.com (C.M.C.A.); ebustamante@saludcastillayleon.es (E.B.M.); 2 Institute of Natural Products and Agrobiology, CSIC-Spanish Research Council, Avda. Astrofísico Fco. Sánchez, 3, 38206 La Laguna, Spain; jm.perezdelalastra@csic.es; 3 Cinquima Institute and Department of Organic Chemistry, Faculty of Sciences, Valladolid University, Paseo de Belén, 7, 47011 Valladolid, Spain; 4 Sistemas de Biotecnología y Recursos Naturales, 47625 Valladolid, Spain; info@glize.eu

**Keywords:** cancer, metallodrugs, cisplatin, structure-activity relationship, ruthenium, photodynamic and photoactivated therapy

## Abstract

This article provides an overview of the development, structure and activity of various metal complexes with anti-cancer activity. Chemical researchers continue to work on the development and synthesis of new molecules that could act as anti-tumor drugs to achieve more favorable therapies. It is therefore important to have information about the various chemotherapeutic substances and their mode of action. This review focuses on metallodrugs that contain a metal as a key structural fragment, with cisplatin paving the way for their chemotherapeutic application. The text also looks at ruthenium complexes, including the therapeutic applications of phosphorescent ruthenium(II) complexes, emphasizing their dual role in therapy and diagnostics. In addition, the antitumor activities of titanium and gold derivatives, their side effects, and ongoing research to improve their efficacy and reduce adverse effects are discussed. Metallization of host defense peptides (HDPs) with various metal ions is also highlighted as a strategy that significantly enhances their anticancer activity by broadening their mechanisms of action.

## 1. Introduction

According to WHO data, cancer is one of the leading causes of death in the world, with almost 10 million deaths in 2020. Globally, the number of diagnosed cases is increasing every year, with a total of 30.2 million new cases expected to be diagnosed by 2040 [1]. Cancer treatments include medical surgery, radiation therapy, hormone therapy and stimulation of our natural defences, but one of the most effective methods is chemical therapy or chemotherapy. Chemotherapy is used to treat numerous types of cancer by stopping or slowing the growth of rapidly growing and dividing cells [2]. Initially, chemotherapy was based solely on organic compounds, but a series of events led to the discovery of the antineoplastic properties of various metal complexes, the first being platinum [3].

The use of platinum complexes is the most classic case of transition metal chemotherapy in cancer treatment, mainly cisplatin. However, there are transition metal complexes other than platinum that may have different antitumour activity and toxic side effects than Pt drugs because of their different chemical behaviour, hydrolytic rates and mechanisms of action [4,5,6,7].

At present, the search continues for compounds that are more effective and have fewer side effects. Studies have been carried out on all the groups of metals in the periodic table, with ruthenium, titanium and gold complexes providing the best results, after platinum complexes, which are currently the most widely used in the clinic and therefore continue to be of great interest [8,9].

**Figure 1:**
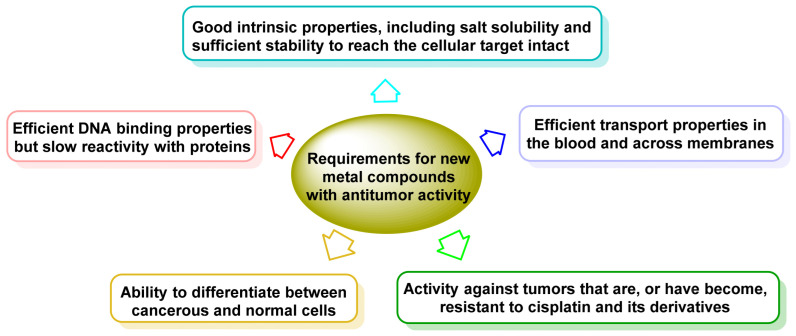
Requirements for new metal compounds with antitumor activity.

The design of new metal DNA-binding compounds with anti-tumour activity and clinical efficacy must meet the following requirements, (Figure 1).

Applied in this field, transition metal complexes showed encouraging perspectives [10,11,12,13,14,15,16,17,18,19]. In this review, we aim to provide a comprehensive examination of the platinum and non-platinum metallodrugs used in cancer therapy, focusing on their molecular mechanisms of action. Particular emphasis is placed on the promising therapeutic possibilities offered by the combination of cisplatin with polyphenols. In addition, we explore phosphorescent Ru(II) complexes that exhibit unique anticancer properties through mechanisms other than cisplatin. Furthermore, we delve into the synergistic potential of metallization of host defense peptides (HDPs) with different metal ions. This innovative strategy holds promise for enhancing the anticancer activity of peptides by expanding their mechanistic capabilities, thus opening new avenues for effective cancer treatments. Metal ions such as copper, platinum, ruthenium, titanium and gold have been investigated for their ability to enhance the therapeutic properties of HDPs. In combination with HDPs, which can disrupt cancer cell membranes and modulate the immune response, these therapies can work synergistically to enhance the anticancer effect. For example, the cytotoxicity of piscidins on cancer cells was enhanced by complexation with Cu^2+^ This phenomenon was explained by a complex description of the interactions between peptides and lipids [20]. By utilizing the intrinsic properties of peptides and metals, this approach offers a versatile solution for cancer treatment and has the potential to overcome challenges such as drug resistance and adverse side effects.

The bibliography consulted was carried out using the PubMed, Scopus and Web of Science databases. We started the search with articles published between 2022 and 2023. The search terms included “cancer” “metallopharmaceuticals”, “use of metallopharmaceuticals for cancer treatment” “metal-based drugs”, “cisplatin”, “ruthenium complexes” and “gold complexes”, including the term “nanotechnology” in the search, and references found in these articles from previous years were reviewed.

## 2. Platinum (II) Complexes

Platinum derivatives release two valences of the platinum ion upon intracellular activation to form two stable bonds with DNA components, resulting in an alteration of the three-dimensional configuration of DNA, and thus the impossibility of strand separation for replication, as well as errors in transcription [21].

### 2.1. Cis-Platinum(II) Complexes

Cisplatin was the first platinum compound approved by the FDA (Food and Drug Administration) for use in clinical therapy, as well as the first metal compound to be approved as an antitumour drug. The first data on cis-platinum date back to the 19th century, when it was first described, but it was not until the 1970s that its anticancer action was discovered.

**Figure 2:**
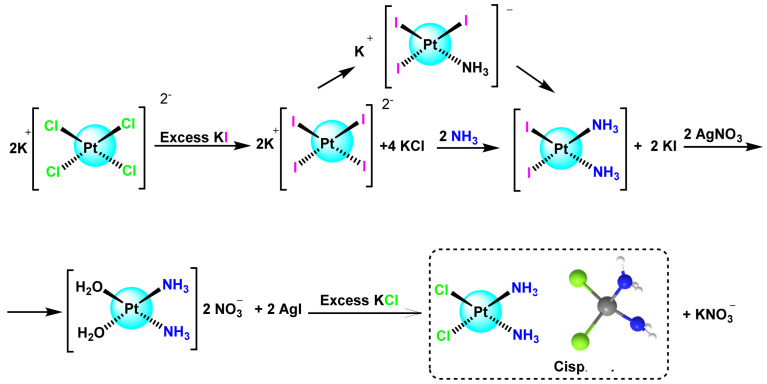
Synthesis of cisplatin by the Dhara method.

The most efficient method for the synthesis of cisplatin was proposed by Dhara, 1979 [22], which was published in 1970 under the title “A rapid method for the synthesis of cis-[PtCl_2_(NH_3_)_2_]” (Figure 2).

Cisplatin (CDDP, cisdiamminedichloroplatinum II, cisplatinum) is a square-planar complex containing two labile chlorine and two relatively inert ammonia molecules coordinated to the central Pt(II) atom in a cis-configuration [23]. The chloride groups are displaced first by water and then by other groups including sites on DNA. Cisplatin can also cross-link proteins to DNA. Although, strictly speaking, the coordination of Pt with DNA bases cannot be considered an alkylation reaction, cisplatin and its analogues are usually studied among the alkylating agents because of the electrophilicity of the active species [24].

Cisplatin is used to treat a variety of cancers such as ovarian, testicular, small and non-small cell lung, breast, head and neck, stomach, oesophageal, cervical, prostate, Hodgkin’s and non-Hodgkin’s lymphomas, melanomas, sarcomas, neuroblastomas, multiple myeloma and mesothelioma, among others. Around US$2 billion worth of platinum-based cancer drugs are sold worldwide [25,26] and almost 50% of all patients are treated with cisplatin [27]. Treatment is limited by the side effects of cisplatin, which produces collateral damage to the patient’s body due to its cytotoxic action on healthy cells, resulting in several unwanted side effects [28,29,30].

**Figure 3:**
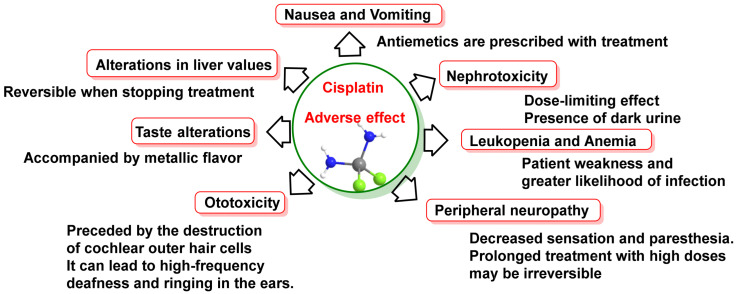
Most relevant adverse effects.

Importantly, the severity of side effects depends on the dose administered, and there is no relationship between the severity of side effects and anti-neoplastic effectiveness. In addition, not all side effects are always experienced and they are usually largely reversible [31]. In descending order of incidence, the most relevant, but not all, adverse effects are listed in Figure 3.

Cisplatin binding to DNA causes a distortion of the helical structure and results in inhibition of DNA replication and transcription [32]. This complex is initially inactive, and it is not until it enters the cell and undergoes an actuation reaction that it is able to carry out its biological effect. After administration of the drug, cisplatin enters the cell by diffusion through the membrane, and once inside, a chlorine atom is displaced by a water molecule. The species resulting from replacing the chloride ligands with water molecules has two positive charges, and is therefore strongly attracted to the phosphate groups that form part of the DNA strands [33]. The affinity between cisplatin and nitrogenous bases is particularly strong for guanine, to which it binds covalently via the N7 of the imidazole ring. The interaction between cisplatin and the nitrogenous bases first results in the formation of a mono-duct and then in the platinum cross-linking with two nitrogenic DNA bases—guanine (G) and/or adenine (A)—and the displacement of the second chlorine atom. Complexation with N-7 of two neighbouring guanine units displaces the two water molecules and leads to intrachain cross-linking [34].

**Figure 4:**
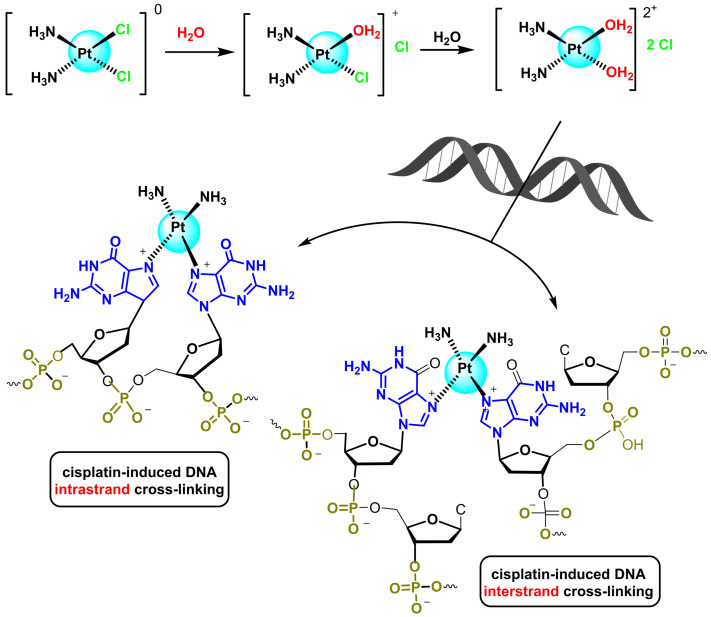
Reaction between DNA and cisplatin.

Cisplatin-induced cross-linking can also occur between two opposing DNA strands. This binding at the N-7 position of the nitrogenous bases causes a twist in the strand, blocking replication and cell division, leading to cell death [35,36,37], (Figure 4).

**Figure 5:**
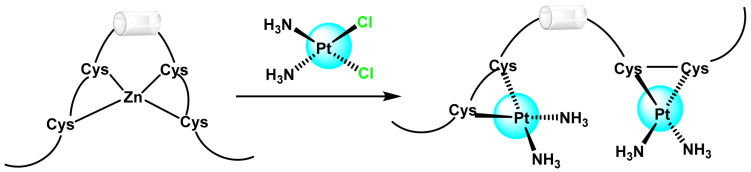
Zinc-finger protein transcription factor coordinated to Zn left and Disrupted conformation of Zn-finger protein transcriptión factor (right figure).

The formation of the initial monoadduct does not always lead to intra- or inter-strand crossover, there is also the possibility of a DNA-cisplatin-protein adduct forming, due to the attraction between the activated drug and the thiol groups of proteins normally found in close connection with DNA. This impedes the processes of DNA replication or repair, contributing to cell death [23,24]. An additional mechanism for the prevention of DNA transcription is the substitution of Zn for Pt in the zinc-finger protein transcription factor [38], (Figure 5).

The zinc cation is essential for coordinating the protein’s amino acids, usually cysteine and histidine, packaging the DNA-binding domains into a dense structure [39]. Replacing the zinc ion with platinum alters this conformation and binds the zinc finger permanently to DNA polymerase-α, which is a transcription enzyme vital for cell replication [40].

**Figure 6:**
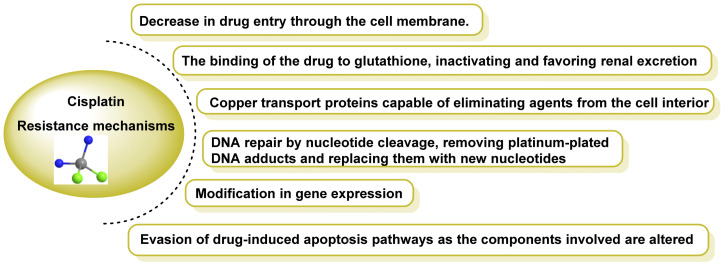
Summary mechanisms of resistance to cisplatin.

Over time, cells develop mechanisms of resistance to toxins or drugs, a phenomenon known as “Multidrug Resistance” (MDR) [41]. The mechanisms of resistance to cisplatin are due to the high affinity of platinum complexes for molecules with thiol groups, such as glutathione or metallothionein [42]. In addition, once DNA adducts have been formed, other mechanisms come into play related to DNA repair, largely regulated by the P53 gene, which has tumour suppressor capacity by triggering apoptosis of abnormal cells, and by signalling cascades of the mitogen-activated proteinase pathways [43]. The most important and best-known resistance mechanisms are shown in Figure 6.

Due to the high toxicity of cisplatin and the problem of intrinsic or acquired drug resistance, many analogue compounds have been developed in an effort to improve its selectivity [44]. The development of cisplatin analogues has revealed requirements that are necessary for their use as an anticancer drug: (i) electroneutrality to allow the drug to cross cell membranes, even if the active form is charged after ligand exchange, (ii) the presence of at least two good leaving groups, preferably cis with each other (iii) the presence of “inert” transporting ligands, usually non-tertiary amine groups that increase the stability of the adduct through hydrogen bonding with nearby bases [45]. To minimise side effects and resistance to cisplatin, combination therapies are used and have been shown to be more effective in combating cancers [23].

**Figure 7:**
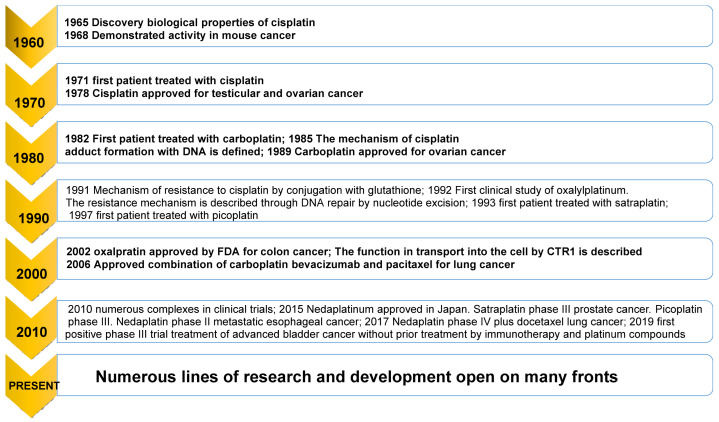
Advances in the field of platinum complexes chronologically ordered, from the discovery of cisplatin to the present.

Platinum compounds were prepared that even exhibited activity against cisplatin-resistant cancers. Figure 7 shows the time evolution in the development and approval of different platinum drugs.

**Figure 8:**
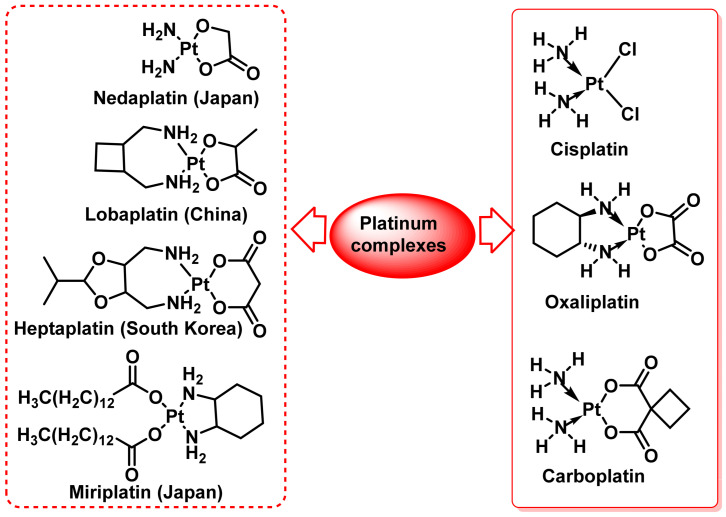
Pt(II) complexes approved worldwide ((**right**) figure) and in some countries ((**left**) figure). All of them have a square-planar geometry, with a coordination index of 4 for Pt.

The need to modify the chemical structure of cisplatin to reduce its side effects led to the synthesis of other cisplatin analogues, some of which have been approved by the FDA and subsequently globally (carboplatin and oxaliplatin), while others are used in specific countries (lobaplatin in China, heptaplatin in South Korea and nedaplatin in Japan) [46], (Figure 8).

#### 2.1.1. Carboplatin

The second-generation platinum drug, carboplatin (cis-diamine (1,1-cyclobutane dicarboxylate) platinum(II) was developed to reduce the dose-limiting toxicity of cisplatin. Carboplatin’s mechanism of action is like that of cisplatin, forming cross-links. It has a structure that differentiates it from cis-platinum, having a bidentate cyclobutane-1,1-dicarboxylate ligand, which together with the existence of steric hindrance caused by the cyclobutane ring, results in a reduced chemical reactivity compared to that of cisplatin [47]. Carboplatin is used to treat ovarian, head and neck, breast, testicular, small cell lung, brain, bladder, cervical and other cancers [48].

It has lower cytotoxicity and nephrotoxicity than cisplatin. It is also less neurotoxic, although adverse reactions related to neurotoxicity increase greatly with age, which is why it is more commonly used to treat paediatric neuroblastomas. Hearing loss typical of cisplatin treatment is not seen. The main adverse effect limiting the maximum dose to be administered is thrombocytopenia, which has been shown to be highly variable between patients, due to individual patient characteristics [29,49].

Carboplatin shows a lower excretion rate than cisplatin, so its effect is longer lasting, and less toxicity to the kidneys, but has negative effects on the bone marrow leading to myelosuppression [50].

#### 2.1.2. Oxaliplatin

Oxaliplatin was approved 2002 by the FDA, and today is used worldwide. It is a platinum complex with (1R,2R)-1,2-diaminocyclohexane (DACH) ligand and oxalate as leaving group and is a third-generation platinum compound. Its main cellular target is DNA, where it acts similarly to cisplatin and carboplatin [51]. Oxaliplatin mainly forms cross-links at adjacent guanine bases or between guanine and adenine, although to a lesser extent [52].

Oxaliplatin is quite effective in combination therapy with 5-fluroacyl for the treatment of metastatic colorectal cancer. The mechanisms of resistance developed by tumour cells are like those described for cisplatin [53]. One of the most effective ways to increase the sensitivity of tumours to oxaliplatin is to administer it in combination with other drugs rather than as a single drug [54].

**Figure 9:**
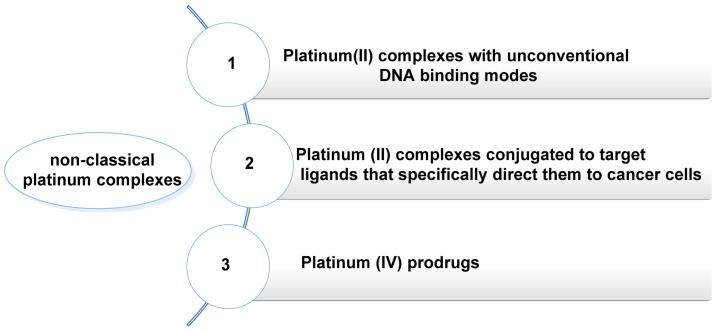
Design of new platinum drugs.

The treatment of cancer with cisplatin and the derivatives described above causes significant side effects, most notably its high nephrotoxicity, and the problems arising from its non-specific cytotoxicity, proven efficacy in few solid tumours and increasing resistance to it. In view of these limitations, other types of platinum complexes are being studied, aimed at increasing selectivity, improving bioavailability and being able to reduce the toxicity of treatment for patients undergoing chemotherapy [55] (Figure 9).

### 2.2. Trans-Platinum(II) Complexes

In 1844, Reiset [56] described for the first time a synthetic procedure for transplatin and, for this reason, it is known as Reiset’s second chloride [57].

Trans isomers of the various complexes, such as transplatin (trans isomer of cisplatin) were initially considered to be ineffective in chemotherapeutic treatment, but this was later shown not to be the case and many trans complexes with cytotoxic activity can be found, which we can classify according to the nature of the ligands [58,59].

#### 2.2.1. Trans-[PtCl_2_L_2_] Complexes where L Are Aromatic Amines

Farrell et al. thought that substitution of NH_3_ in trans-[PtCl_2_(NH_3_)_2_] by sterically hindered ligands could lead to a reduction in the displacement constant of chloride ligands by aquo ligands and perhaps thereby increase their cytotoxicity [60].

**Figure 10:**
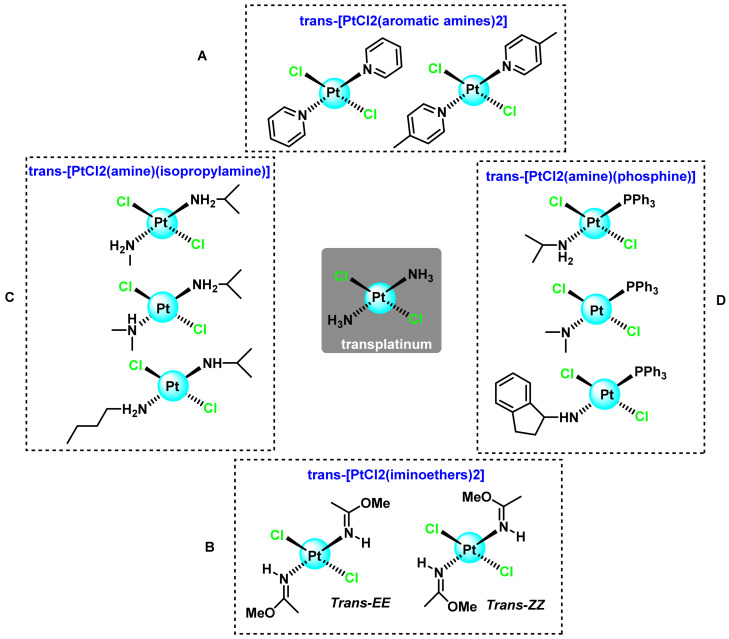
(**A**) Trans-[PtCl_2_L_2_] complexes where L are aromatic amines; (**B**) trans-[PtCl_2_L_2_] complexes where L are iminoethers; (**C**) trans-[PtCl_2_L_2_] complexes where L are aliphatic amines and (**D**) trans-[PtCl_2_(triphenylphosphine)(aliphatic amine)] complexes.

Several platinum(II) complexes with trans geometry and aromatic planar amines were synthesised, and the complexes studied were 10 times more active than transplatin, with cytotoxicities almost equivalent to that of their cis-isomers and even, in some cases, to that of cisplatin [61]. The first complexes to show greater cytotoxicity than their cis-isomers were those where L is a pyridine or a 4-methylpyridine, (Figure 10A).

#### 2.2.2. Trans-[PtCl_2_L_2_] Complexes where L Are Iminoethers

Substitution of an NH3 ligand of transplatin with an iminoether ligand also resulted in a significant increase in cytotoxicity [62]. Trans complexes with two iminoether ligands resulted in increased cytotoxicity, being better than their cis analogues and comparable to cisplatin, and showed antitumour activity in cisplatin-resistant cells [63,64].

The antineoplastic activity of these compounds was also found to be related to the isomerism of the iminoether double bond. Cytotoxic studies in murine P388 leukaemia cells show differences depending on the geometry of the double bond, with the trans-ZZZ complex showing greater cytotoxicity than the trans-EE complex, so the biological mechanism leading to the cytotoxicity of compounds with iminoether ligands with EE [65] and ZZ [62] configurations has been studied in depth (Figure 10B).

The trans-EE iminoether complex forms mainly mono-ducts with guanine residues of DNA and is then able to bind to proteins. This type of adduct is different from those formed by cisplatin and causes increased cytotoxicity by preventing DNA replication (via DNA polymerase) and can prevent double helix repair by inhibiting the removal of this adduct from DNA via NER mechanisms [65]. Trans-ZZZ isomers form adducts with DNA that slowly transfor-man into cross-links between complementary cytosine and guanine residues. These interstrand crosslinks influence the flexibility of the double helix strands and are not recognised by HMGB1.

#### 2.2.3. Trans-[PtCl_2_L_2_] Complexes where L Are Aliphatic Amines

Asymmetric complexes with aliphatic amines of general formula trans-[PtCl2(amine)(isopropylamine)] where the amine can be a methyl, dimethyl and butyl residue, (Figure 10C), are shown to have similar or superior cytotoxicity to cis-platinum in sensitive cells (Jurkat, HeLa, Vero) and resistant (HL-60, Pam 212-ras) to the latter. Comparison of these complexes with their cis-analogues showed that the trans-isomers have greater antitumour activity than the corresponding cis-isomers and that the compound with the best cytotoxicity was the trans-[PtCl_2_(dimethyl) (isopropylamine)]. The cell lines used in the first studies were both sensitive (CH1 and Pam212) and resistant (CH1cisR and Pam 212-ras) to cisplatin [66].

#### 2.2.4. Trans-[PtCl_2_(Triphenylphosphine Aliphatic Amine)] Complexes

Other ligands in trans to the aliphatic amines were the triphenyl phosphines, (Figure 10D), thus attempting to increase the lipophilicity of the complexes to achieve a higher membrane permeability and, consequently, a higher concentration of the complex in the intracellular fluid, resulting in an increase in the probability of interaction with DNA. Thus, it can be assumed that the increased activity is produced by the hydrophobic phosphine ligands phosphine.

Trans platinum(II) complexes possess greater antiproliferative activity than their cis-analogue [67]. The reactivity of trans platinum complexes seems to be related to the structure of the non-salient ligands they may have. These may hinder the formation of intra-strand adducts, leading to a higher proportion of inter-strand bonds being formed, the latter adducts being much more difficult to remove by nucleotide excision DNA repair mechanisms. The discovery that several trans-platinum(II) complexes exhibited antitumour activity, both in vitro and in vivo, against cisplatin-resistant cells, implied differences in the DNA binding of both types of complexes forced a re-evaluation of the structure-activity relationship of these antineoplastic agents [68]. The differences between these two types of geometry are due to the different formation of DNA adducts.

### 2.3. Polynuclear Platinum(II) Compounds

Multinuclear platinum complexes represent a new class of anticancer agents, distinct in terms of DNA binding and antitumour activity profile from their mononuclear counterparts [69,70,71,72]. Polynuclear Pt(II) complexes having trans-[Pt(NH_3_)_2_Cl] units that form bridges with al-canodiamine bonds of varying length are active against cancer. BBR 3464 was selected as one of the best polynuclear Pt(II) for preclinical testing and is active against gastric carcinoma GFX214 and MKN45 in mice [36]. It is a trinuclear platinum(II) complex featuring trans geometry with platinum(II) centres separated by 1,6 hexanediamine connectors and trans labile chloride terminal ligands, which was the first and only platinum(II) drug not based on the “classical” cisplatin structure to enter clinical trials. The characteristics are (i) potency, (ii) ten times lower maximum tolerated dose compared to cisplatin and iii) broad spectrum of tumours sensitive to this agent. For the 4+ loading, the bifunctional binding to DNA where the binding sites are separated by large distances and the consequences of such binding to DNA suggest that this advance alters the paradigm of cisplatin-based anti-tumour agents.

**Figure 11:**
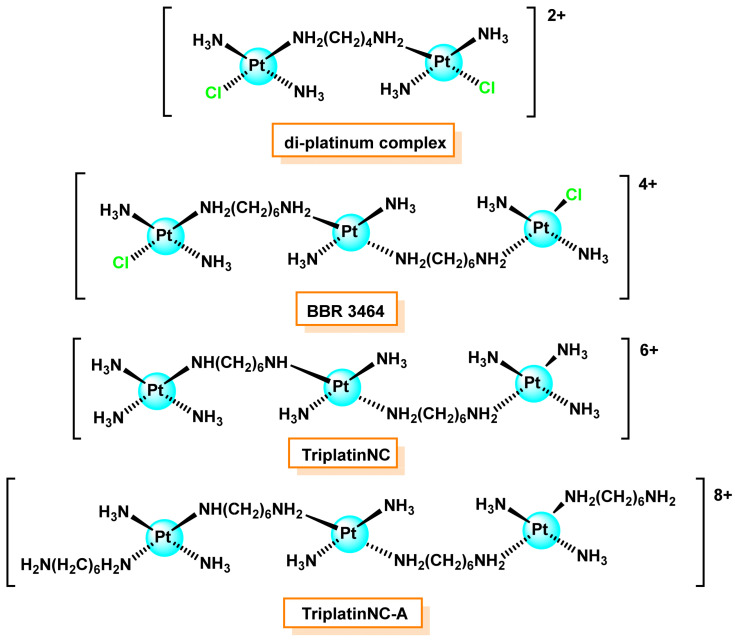
Chemical structures of di and trinuclear Platinum agents.

Summa et al., 2007, demonstrated that the trinuclear complex forms long-range delocalised intra- and intercatenary cross-links between guanines spanning up to six base pairs, resulting in more flexibility and less distortion [73]. Triplatin-NC is another multinuclear Pt(II) complex that avoids deactivation by intracellular nucleophiles and shows improved anti-tumour activity [74], (Figure 11).

The diplatin complex forms 1,2, 1,3 and 1,4 cross-links between guanines on opposite strands. In 1,3 and 1,4 cross-links [75,76], the guanines are separated by one and two base pairs, respectively, while the 1,2 cross-link is formed between guanines of neighbouring base pairs. These unconventional DNA adducts allow the diplatin complex to overcome cisplatin resistance in ovarian cancer cells [71].

## 3. Platinum (IV) Prodrugs

One of the reasons why platinum(II) complexes such as cisplatin and its analogues possess potent activity against different types of cancer is that they include amine-type ligands that give them this antitumoral capacity [77]. However, the coordination sphere conferred by their planar-square geometry makes them more sensitive to ligand substitution, which leads to a certain toxicity.

**Figure 12:**
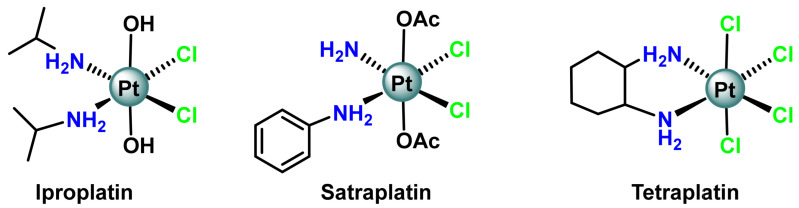
Structures of some examples of anticancer Pt(IV) complexes.

Recently, Pt(IV)-based compounds [78,79,80] are the most promising classes of new generation platinum drugs. Pt(IV) complexes showing anticancer activity are also co-known. Tetraplatin, iproplatin and satraplatin are very important examples of Pt(IV) prodrugs [81], (Figure 12).

**Figure 13:**
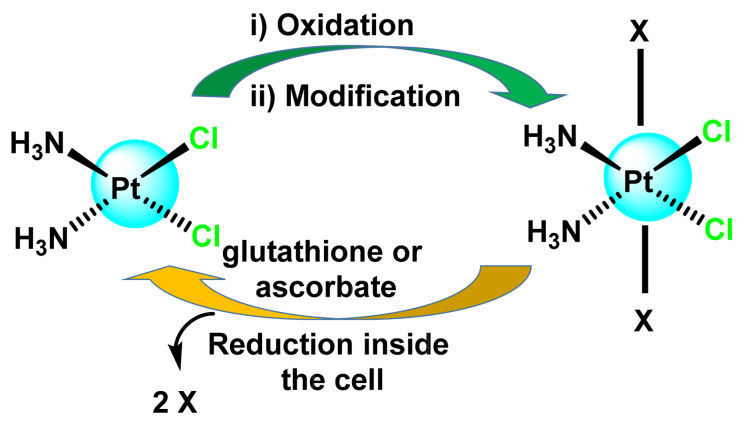
Formation and activation of Pt(IV) prodrugs.

Pt(IV) complexes act as prodrugs and need to be reduced to Pt(II) by intracellular or extracellular reducing agents to show anti-cancer activity [82]. Before binding to DNA, Pt(IV) prodrugs are generally re-duced by glutathione and ascorbate to form square and flat active Pt(II) drugs. The synthesis is performed by oxidation of Pt(II) and thus achieves an octahedral geometry in a way that keeps the ligands of the Pt(II) complex in the equatorial plane and incorporates two new axial coordination positions, (Figure 13), which could lead to an improvement, giving the possibility of oral administration. In contrast, Pt(II) complexes do not have the option of oral administration [83].

Pt(IV) complexes, with a low spin d6 electronic configuration, are kinetically more inert to ligand substitution than Pt(II) complexes, thus reducing their reaction with biomolecules other than DNA and thus reducing side effects. Pt(IV) prodrugs are stable and inert to substitution, which inhibits the reaction with plasma proteins in the blood [37]. The most important feature is that axial groups can be used to increase solubility, lipophilicity, target cancer cells or activate different biological properties. Axial groups can also be conjugated to nanoparticles or other carrier systems for cargo delivery of Pt(IV) prodrugs [84,85].

The design of Pt(IV) prodrugs is another very important way to improve the efficacy of chemotherapeutic drugs in the future. The use of nanoparticle-conjugated Pt(IV) drugs will be a good line of research in the future, as nanoparticles can carry a higher number of Pt(IV) compounds, target cancer cells by binding targeting agents, increase solubility by binding hydrophilic debris, increase distribution to tumour sites and have some other effective advantages [86].

### 3.1. Platinum (IV) Complexes with Bioactive Ligands

**Figure 14:**
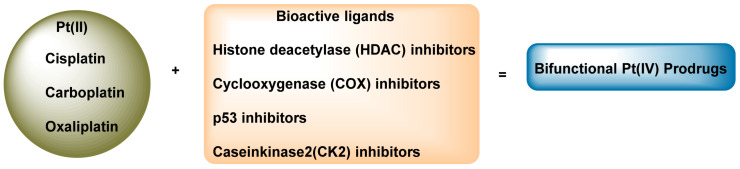
Formation of Pt(IV) prodrugs with various bioactive ligands.

The design of bifunctional Pt(IV) prodrugs is based on the reduction reaction of intracellular Pt(IV) to produce an active platinum(II) species and a biologically active ligand, thus enhancing the accumulation and activity of platinum drugs or helping them to overcome drug resistance [87]. The main Pt(IV) cores currently used are with cisplatin, carboplatin and oxaliplatin, and bioactive ligands include histone deacetylase (HDAC) inhibitors, cyclooxygenase (COX) inhibitors, p53 inhibitors and casein kinase 2 (CK2) inhibitors [37] (Figure 14).

### 3.2. Pt(IV) Prodrugs with Histone Deacetylase (HDAC) Inhibitory Ligands

**Figure 15:**
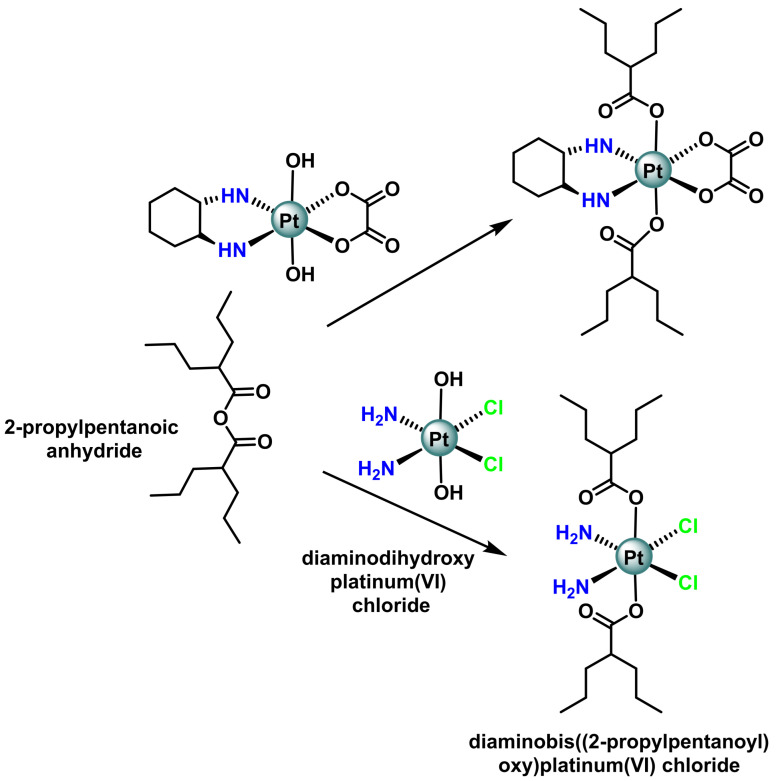
Structure of Pt(IV) prodrugs based on valproic acid.

Yang et al. have designed and synthesised a bifunctional Pt(IV) prodrug, VAAP [88]. Pt(IV) conjugated with valproic acid (VPA) at two axial positions, (Figure 15), binds to DNA to a greater extent than cisplatin. VPA-conjugated Pt(IV) derivatives of cisplatin accumulate and kill cancer cells more effectively than cisplatin due to the significantly higher binding of the platinum-containing VPA to DNA. This superior effect was mainly attributed to increased hydrophobicity. Novohradsky’s team, 2014, synthesised another new bifunctional complex Pt(IV) platinum (IV) derivatives of oxaliplatin conjugated to valproic acid (VPA) [89], a drug that has histone deacetylase inhibitory activity.

**Figure 16:**
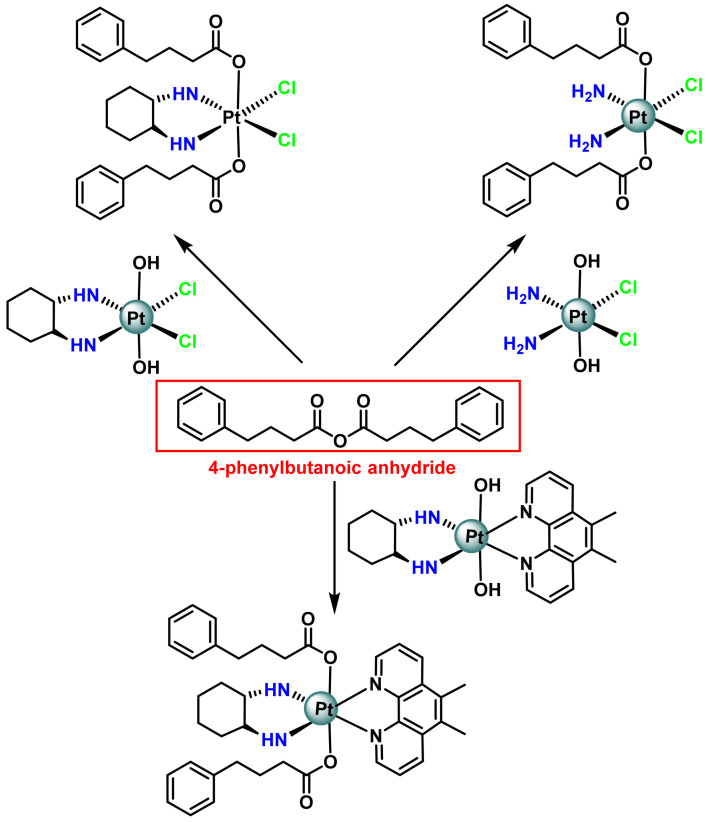
Structures of Pt(IV) prodrugs with 4-phenylbutyric acid ligands.

Another HDAC inhibitor, 4-phenylbutyric acid, Raveendran et al., 2016, synthesised other Pt(IV) derivatives based on the cisplatin and oxaliplatin cores [90], (Figure 16). The Pt(IV) complex with two phenylbutyrate axial ligands is shown to be up to 100 times more effective than cisplatin in many human cancer cells, and they compared its bioactivities with those of VPA-conjugated Pt(IV) derivatives. The researchers found that Pt(IV) derivatives of cisplatin were more potent than those of oxaliplatin. Pt(IV) derivatives of cisplatin with two axial phenylbutyrate ligands are more potent than cisplatin or Pt(IV) derivatives of cisplatin with two hydroxides, two acetates or two valproate ligands [90].

The results of this study suggest that Pt(IV) derivatives of cisplatin with two axial HDACs are superior to those of oxaliplatin, despite the higher potency of oxaliplatin compared to cisplatin. These results also show that the dual-targeting strategy is a valuable route to follow in the design of platinum agents that may be more effective in cancers that are resistant to conventional cisplatin therapy.

**Figure 17:**
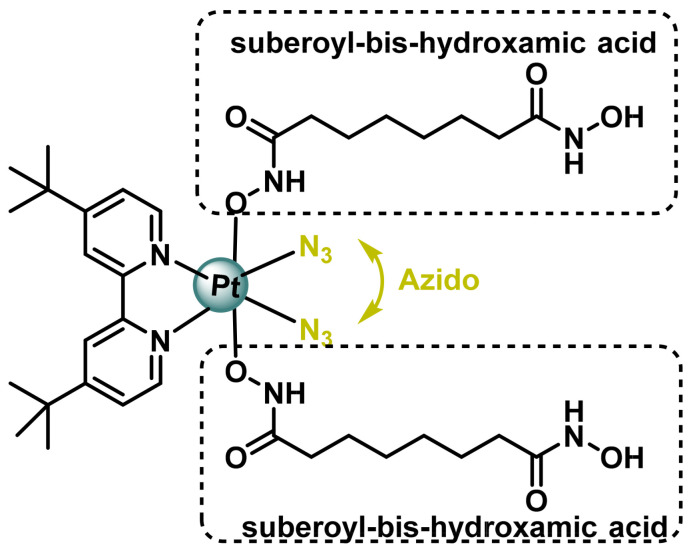
Structure of Pt(IV) prodrugs based on suberoyl-bis-hydroxamic acid.

Suberoil-bis-hydroxamic acid (SubH), (Figure 17), is another compound showing HDAC inhibitory activity. Kasparkova et al., 2015, added two SubH to a non-toxic Pt(IV)-azide complex to form a photoactivatable Pt(IV) complex that showed enhanced efficacy in photodynamic cancer chemotherapy [91]. After irradiation with ultraviolet A (UVA) light, the cytotoxicity of Pt(IV) significantly increased by 6–11 times that of cisplatin, which was unaffected by light. The conjugated complex exerts, after photoactivation, two functions (i) activity as a platinum (II) anticancer drug (ii) inhibitor of histone deacetylase (HDAC) in cancer cells [92].

This new approach is based on the use of a Pt(IV) prodrug, acting through two independent mechanisms of biological action in a synergistic manner, which can be selectively photoactivated to a cytotoxic species in and around a tumour, thus increasing selectivity towards cancer cells [93,94,95].

**Figure 18:**
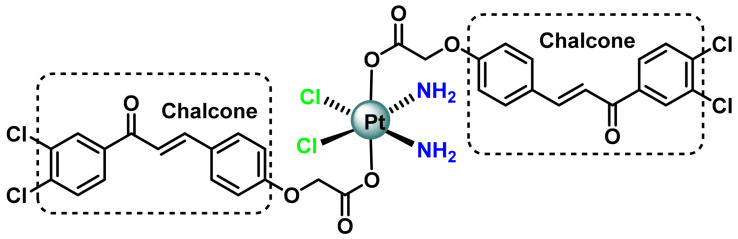
Chalcoplatinum structure.

Chalcoplatin, (Figure 18), is the first example of a Pt(IV) prodrug containing p53 activator residues and was synthesised by Ma et al., 2015 [96]. It is a novel Pt(IV) anticancer prodrug chalcoplatin, based on cisplatin and chalcone, which not only formed Pt-DNA adducts but also activated the p53 pathway. The results showed that, compared to cisplatin, chalcoplatin exhibited markedly higher cytotoxicity (10-fold) for wild-type p53 cells but not for p53 null cells. Although cell accumulation increased, the Pt-DNA binding rate was not improved. Chalcoplatin mainly induced cell cycle arrest in S and G2/M phases, whereas cisplatin and chalcone induced cell cycle arrest in S and G2/M phases, respectively [96].

**Figure 19:**
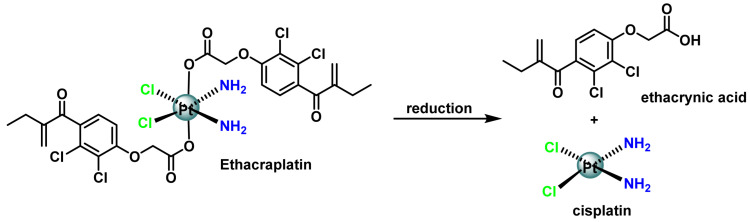
Ethacraplatin reduction.

When reduced, ethacraplatin produces cisplatin and ethacrynic acid [97,98], (Figure 19), which inhibits glutathione S-transferase (GST). As a result, the platinum drug reacts with GSH at a very low rate and therefore drug resistance is significantly decreased, allowing ethacraplatin to inhibit the growth of cisplatin-resistant breast, lung and colon cancer cells more effectively than cisplatin alone [99].

Reduction of the platinum (IV) divalproate (VAAP) complex generates cisplatin and 2 equivalents of valproic acid, a potent histone deacetylase (HDAC) inhibitor that stimulates differentiation and apoptosis in cancer cells [88].

**Figure 20:**
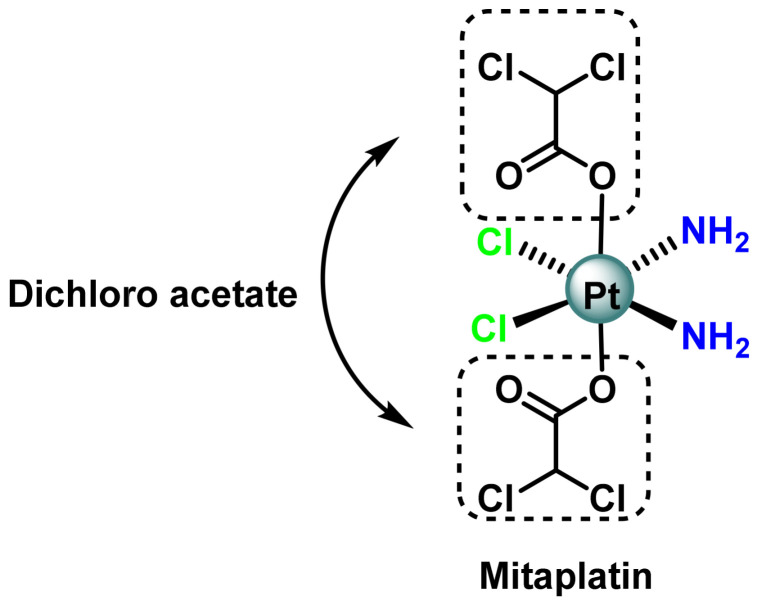
Chemical structure of mitaplatin.

Another Pt(IV) prodrug included in clinical trials is mitaplatin, (Figure 20), which consists of two dichloroacetates (DCA) in axial positions attached to a cisplatin core. Mitaplatin is designed to selectively kill cancer cells rather than non-malignant cells [100]. Following cancer cell depletion, DCA inhibits pyruvate dehydrogenase kinase (PDK), which in turn reduces the flow of metabolites through aerobic glycolysis and restores normal myocardial function. This process promotes apoptosis through the release of cytochrome c from mitochondria and translocation of apoptosis-inducing factor (AIF) to the nucleus [101]. At the same time, free cisplatin induces DNA damage in the usual way and causes cell death by apoptosis. The concerted action of cisplatin and DCA allows mitaplatin to destroy lung carcinoma cells (A549) more readily than normal lung fibroblasts (MRC5) in in vitro cultured systems.

**Figure 21:**
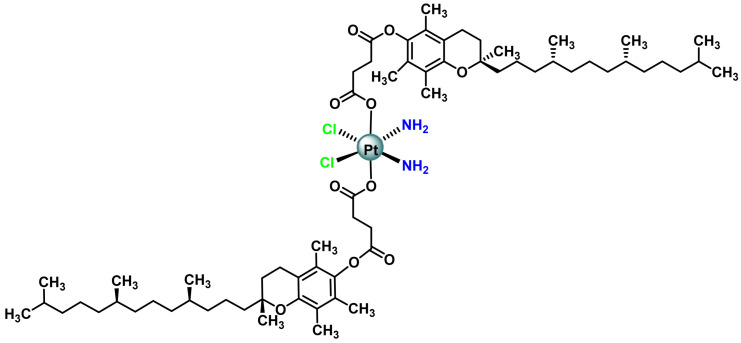
Chemical structure of Pt(IV) complex containing vitamin E derivatives.

Other Pt(IV) complexes synthesised contain cisplatin bound to one or two α-tocopheryl succinate ligands (a vitamin E analogue), (Figure 21). In this derivative, the disubstituted derivative was found to be non-toxic, while the monosubstituted derivative showed 7–25 times the cytotoxicity of cisplatin in several tumour cell lines [102].

The incorporation of two axially coordinated estrogen moieties into a cisplatin prodrug allowed the preparation of cisplatin-estrogen conjugates capable of releasing both groups simultaneously. Oestradiol activity enhances cisplatin activity. As the oestrogen units were modified with ester groups, hydrolysis to generate free oestradiol is a prerequisite for the activity of cisplatin [103].

## 4. Plant Extract-Cisplatin Combination

At the clinical level, cisplatin is administered together with other drugs in order to enhance their properties. Another possibility could be the co-administration of cisplatin with plant extracts. In preclinical studies, natural products have been shown to enhance the therapeutic activity of cisplatin and attenuate its chemotherapy-induced toxicity [104,105,106,107].

Polyphenols may act preventively or after transformation has taken place. The therapeutic potential of polyphenols is based on their antioxidant, anti-inflammatory and prebiotic effect [108], as they can contribute to the reduction of the pro-oxidant and pro-inflammatory environment required by the tumour mass for its proliferation [109], as well as correcting a situation of imbalance in the microbial balance in the microbiota (dysbiosis). If the alteration of the tumour microenvironment is possible, it could provoke the cessation of tumour growth, which would lead to an improvement in the therapeutic effect of adjuvant chemotherapy [110]. Moreover, plant extracts rich in polyphenols may be able to induce tumour cell death directly by acting on various molecular targets [111,112,113,114].

**Figure 22:**
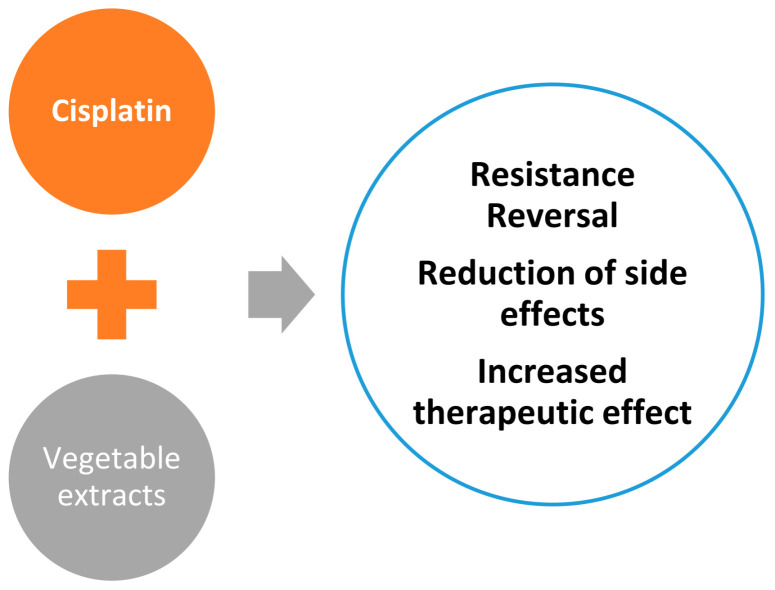
Beneficial effects of using plant extracts together with cisplatin.

There are three possibilities in the use of cisplatin with plant extracts: (i) increasing sensitivity to cisplatin in cell lines that did not respond to treatment; (ii) improving the living conditions of patients undergoing chemotherapeutic treatments; and (iii) the use of plant extracts to increase the therapeutic effect of cisplatin on tumours that are not resistant to this compound, (Figure 22).

**Figure 23:**
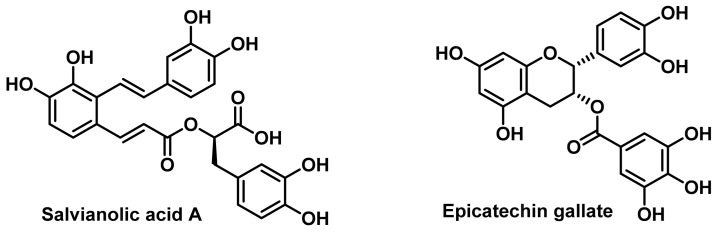
Chemical structure of salvianolic acid A and Epicatechin gallate.

With the use of plant extracts, it has been possible to restore sensitivity to cisplatin in cell lines that did not respond to treatment, such as the A549/DPP lung cancer cell line. Treatment of this tumour model with a mixture of cisplatin and salvianolic acid A or epicatechin-3-gallate can re-sensitise the cells to the therapy [115], (Figure 23).

Sharma et al., 2017, found that hydroalcoholic extract of *Glycyrrhiza glabra* (liquorice) and quercetin reversed cisplatin resistance in MDA-MB-468 triple-negative breast cancer cells by inhibiting the enzyme cytochrome P450 1B1. Using enzyme activity determinations and bioinformatics assays, they concluded that quercetin was the bioactive compound most likely to be responsible for the observed effect [116].

The use of cisplatin is limited by the damage it causes, as it is a very powerful chemotherapeutic agent, and research is being carried out to reduce its side effects on healthy tissues without altering its anti-tumour effect [117]. The use of plant extracts in clinical practice could contribute to an improvement in the living conditions of patients undergoing chemotherapeutic treatments, as it has been observed that they can reduce their negative effects [118]. Cisplatin treatment decreases the activity of antioxidant enzymes such as superoxide dismutase and catalase, and the administration of plant extracts can reverse this, protecting tissues against oxidative damage [119].

Other authors suggest that the anti-inflammatory effect of plant extracts may also play a key role in this protective role by reducing cisplatin-induced renal and hepatic side effects in rats through the antioxidant and anti-inflammatory properties of *Malva sylvestris L.* extract [120].

The protective effect of plant extracts against cisplatin damage can be extended to other areas of the body such as the immune system [121], the testicles [122,123] and the heart [124,125]. Pre-clinical tests on animals have shown that ginger extract can be used to treat chemotherapy-induced nausea and vomiting; however, the results of research in clinical trials have been inconclusive [126]. Daily consumption of 160 mg of ginger may have beneficial properties in controlling cisplatin-induced nausea in women and in head and neck cancer patients [127]. These studies have been conducted in animals and have not yet been validated in human patients.

The third aspect to consider would be the use of plant extracts to increase the therapeutic effect of cisplatin on tumours that are not resistant to this compound. The aim is to achieve an additive or synergistic effect between the two drugs due to their different therapeutic targets [128]. The analysis of the effect of a hibiscus flower extract on the MDA-MB-231 cell line was studied by Nguyen et al., 2019, as a model of triple-negative breast cancer, one of the subtypes with the worst prognosis. The authors observed that this extract was able to induce cell death by apoptosis, the process being mediated by an increase in oxidative stress. They then co-administered this extract at a fixed concentration with varying concentrations of cisplatin, and observed an increased therapeutic effect compared to the use of the metallic drug alone [129].

**Figure 24:**
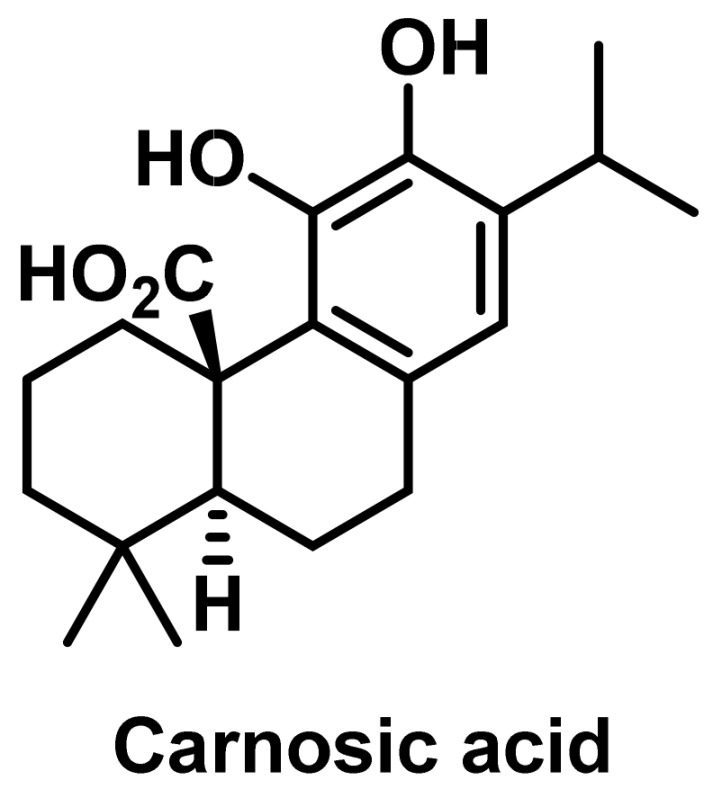
Chemical structure of carnosic acid.

Carnosic acid, (Figure 24), a polyphenolic diterpene isolated from rosemary (*Rosemarinus officinalis*), has also been reported to have several pharmacological and biological activities. The combined effect of cisplatin plus carnosic acid has been studied in mouse Lewis lung cancer xenografts. The combination of carnosic acid and cisplatin produced significantly better anti-growth and proapoptotic effects than the drugs alone. Carnosic acid promoted CD8+ T-cell lethality, which contributed to the enhanced effect of cisplatin against lung cancer [130].

## 5. Combining Metallodrugs with Host Defense Peptides for Enhanced Anticancer Therapy

In recent years, oncology has made significant progress by integrating different therapeutic strategies to improve efficacy and minimize toxicity. Among these strategies, the combination of metallodrugs with host defense peptides (HDPs) has emerged as a promising approach in the fight against cancer. Metallodrugs, which are known for their potent anticancer properties, often act via mechanisms such as DNA binding, protein interaction and induction of oxidative stress.

### 5.1. Combining Metallodrugs with HDPs

**Figure 25:**
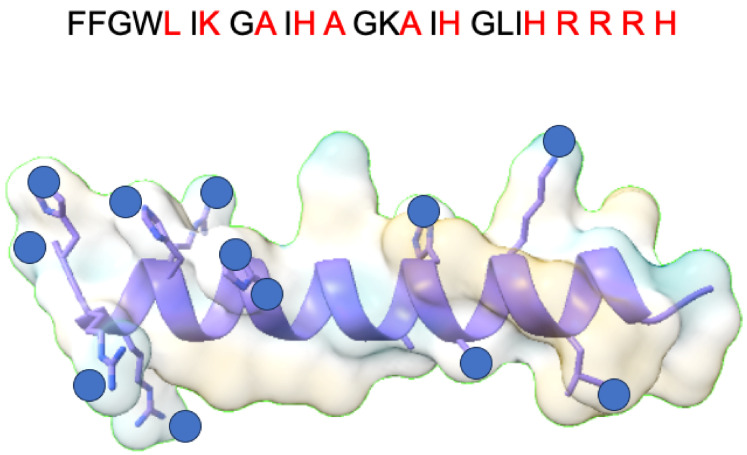
Computational prediction of the binding sites of Zn^2+^ (residue highlighted in red) to the HDP Chrysophsin-1 from *Pagrus major* (Red seabream) using IonCom server (https://zhanggroup.org/IonCom/ioncomsubmit.cgi), accessed on 5 June 2024. Peptide structure was visualized using ChimeraX (version 1.6.1).

The metallodrug is bound to the peptide either on a solid carrier or in solution after separation of the peptide from the resin. The peptide carrier is usually prepared by solid phase synthesis. A linker with the ability to chelate metals can be incorporated into the peptide sequence, or the metal can be bound directly via a suitable amino acid. The direct binding of the metal to the peptide or the indirect binding via suitable linkers and the subsequent metallization of the peptide make the conjugation schemes as flexible as the metal–peptide bioconjugates [131]. The diverse chemical and structural properties of transition metal complexes offer many innovative approaches for the development of compounds that can have precise chemical or physical effects on specific biological entities [132]. Site-selective generation of advanced metal-peptide conjugates is often challenging because peptides have multiple donor atoms that can coordinate to metal centers. Determining the exact sites where peptides bind to ions is crucial for deciphering how peptides work and for developing new drugs. Computational prediction of metal and acid radical ion binding sites can be used to determine peptide ion binding sites [133] (Figure 25).

In a study by Truong et al., a transmetalation-based synthetic solid support approach is presented using an imidazolium pro-ligand to selectively anchor various transition metal half-sandwich complexes to peptides despite the presence of multiple coordinative motifs. Although the resulting metallized peptides did not exhibit cytotoxic activity against human cancer cell lines, this platform shows significant potential for site-selective peptide labelling, biorthogonal applications, and novel ligand design strategies for transition metal catalysts [134].

### 5.2. Piscidins with Cu^2^⁺

Piscidins, host defense peptides (HDPs) from fish, have a number of beneficial properties, including antimicrobial, antiviral, anticancer, anti-inflammatory and wound-healing effects. Piscidins are characterized by their high histidine content. Due to a conserved histidine at position 3, they have an amino-terminal copper and nickel binding motif (ATCUN). The ATCUN motif offers innovative approaches for the treatment of various diseases, including cancer [135]. Metallization of these peptides reduces their overall charge and introduces a redox center capable of generating radicals that convert unsaturated fatty acids (UFAs) into membrane-stabilizing oxidized phospholipids (OxPLs). Upon metallization, the ATCUN motif not only becomes planar, but it also releases several protons from its three nitrogen ligands in the backbone. This leads to the formation of non-protonated amides at positions 2 and 3 [136].This metallization process enhances the membrane disrupting effect of Piscidin 1 (P1) and increases its specificity for the anionic lipids present on pathogenic cell membranes, optimizing both its antimicrobial efficacy and its targeting effect in the presence of OxPLs [137]. A study conducted by Comert et al. has shown that the incorporation of metal ions into piscidins, dramatically increases their anti-cancer efficacy by causing physical and chemical damage to lipid membranes. Piscidins 1 and 3 (P1/3) are histidine-rich, amphipathic host defense peptides (HDPs) that interact with membranes and penetrate cells. These peptides adopt an α-helical conformation when they bind to membranes. While P1-apo is more potent than P3-apo, its cytotoxicity is increased by a factor of two or seven by metallization. In particular, P3 shows remarkable efficacy in incorporating its metallized pattern into the bilayers, leading to the formation of water gaps within the hydrocarbon region and the strategic placement of Cu^2^⁺ ions near the double bonds of the acyl chains, promoting their oxidation [20]. These results suggest that metallization of HDPs can improve their mechanical capabilities, potentially leading to the development of more powerful peptide-based anticancer agents.

### 5.3. Cisplatin and Tachyplesin I

In vitro experiments have shown that the combined use of tachyplesin I, from the hemocytes of the horseshoe crab *Tachypleus tridentatus*, and cisplatin can significantly increase anticancer efficacy while reducing the required dose of cisplatin. This combination allows a reduction in the effective dose of cisplatin, minimizing its non-specific toxicity. Tachyplesin I increases the permeability of cancer cell membranes, facilitating the uptake and effect of cisplatin. This synergistic interaction not only improves the overall anticancer effect, but also mitigates the undesirable side effects typically associated with high-dose cisplatin therapy [138,139].

### 5.4. Combined Cytotoxic Effects

In a novel approach to metal bioconjugation, researchers observed an enhancement of antiproliferative activity by the conjugation of organometallic ruthenium (Ru) and osmium (Os) anticancer agents with a peptide carrier, in contrast to the typical reduction in such conjugations. A study reports the first organometallic Ru half-sandwich bioconjugate exhibiting significant antiproliferative activity in the low micromolar range against CH1 ovarian cancer cells, while the non-metallized peptide 4 showed no activity [140]. This finding underscores the potential of this bioconjugation strategy to improve the efficacy of organometallic anticancer agents and open new avenues for therapeutic development.

## 6. Metallocene Complexes in Cancer Therapies

**Figure 26:**
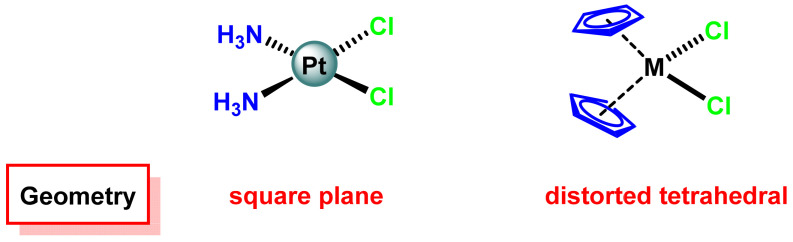
Chemical structure of cisplatin and metallocene dichloride.

The mechanism of action of cisplatin is directly related to the presence of two cis-positioned chloride ligands, and with this in mind much research focused on the synthesis of new compounds with labile cis-positioned ligands using metals that could be less toxic [141]. The metallocene dichloride type derivatives received special attention mainly due to the structural similarity with the cisplatin complex due to the existence of two chloride ligands in the cis position, (Figure 26).

In metallocenes two ligands are cyclopentadienyl bonded to the metal in a pentahaphatic manner, and the other two are chloride ligands, which are labile enough to be hydrolysed on contact with the biological environment.

Petra Köpf-Maier’s group, 1979, evaluated the in vivo cytotoxic activities of a series of metallocene complexes using different transition metals and found that the derivative with the highest biological activity was the titanocene dichloride complex [142].

### 6.1. Titanocene Dichloride

**Figure 27:**
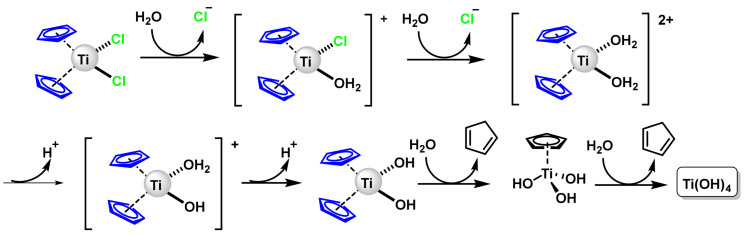
Hydrolysis of the titanocene dichloride complex.

Titanium has several oxidation states, although only the tetravalent compounds (Ti^4+^) are of interest as antitumour agents. Titanocene dihalides (titanocene dichloride and titanocene “Y”) are titanium(IV) derivatives, which are less toxic than Pt(II) derivatives. The cytotoxicity of Cp_2_TiCl_2_ is based on the rapid hydrolysis of the compound in aqueous solution. This compound is poorly soluble in water, but with the aid of a co-solvent such as DMSO, the chloro ligands dissociate rapidly [143]. At acidic pH (3) the first chlorine ligand dissociates within seconds and the second chlorine ligand dissociates with a half-life of fifty minutes. This dissociation process is accompanied by acylation and hydrolysis. The cyclopentadienyl rings dissociate with a half-life of 57 h. and when hydrolysed form titanium hydroxide which is inert [144], (Figure 27).

Although titanocene dichloride does not cause kidney damage, which is why it has been used in kidney and breast cancer, it is no longer used because it is very unstable and shows little efficacy.

A total of five clinical trials were conducted to examine Cp_2_TiCl_2_. Three phase I trials involved patients with advanced solid tumours and evaluated the dose-limiting toxicity of the compound. Cp_2_TiCl_2_ advanced to phase II testing. Despite promising in vitro data, none of the patients admitted to the phase II trials [145,146] achieved a complete or partial response to Cp_2_TiCl_2_ therapy. Failure in Phase II trials resulted in the compound being eliminated from further testing. The hydrolytic instability of Cp_2_TiCl_2_ may be a major obstacle to its therapeutic potential [144].

### 6.2. Titanocene Y

**Figure 28:**
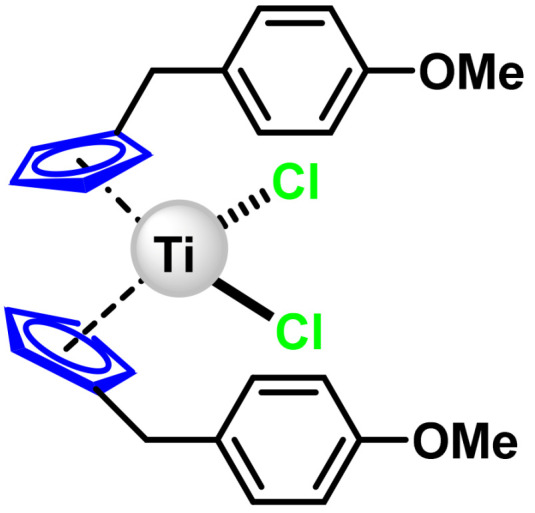
Chemical structure of bis-[(p-methoxybenzyl)cyclopentadienyl]titanium dichloride (Titanocene Y).

Titanocene Y, (Figure 28), another titanocene dihalide, induces apoptosis in solid tumours such as leukaemia and lymphomas, and together with vincristine has been found to provide a good synergistic effect, being able to prevent cancer cell resistance. Studies on its mechanism of action are limited, it is thought to bind to phosphate groups in DNA. Titanocene Y is very stable in water with a half-life of more than a week [147].

### 6.3. Budotitane

**Figure 29:**
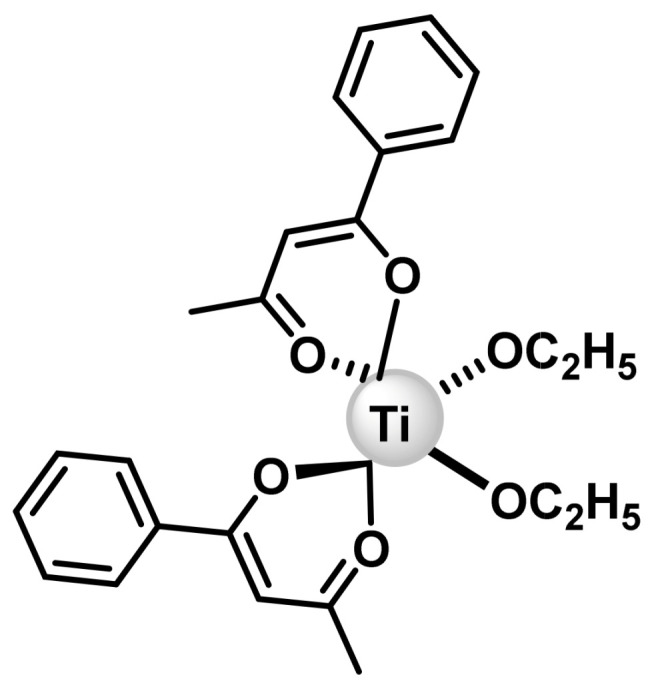
Chemical structure of [cis-diethoxybis(1-phenylbutane-1,3-dionato) titanium (IV)] (Budotitane).

Budotitanium has been shown to be active against cisplatin-resistant solid tumours, however, its use is limited by liver damage [148]. Budotitanium was the first Ti(IV) compound used in clinical trials. Two phase 1 trials were conducted in cancer patients unresponsive to other treatments. In the first trial, the dose-limiting toxicity was nephrotoxicity (accompanied by nausea, vomiting, weakness and malaise) [149], and in the second was cardiac arrhythmia [148]. In both studies, some patients experienced an altered sense of taste. No tumour response to butdotitanium was observed in either study or the compound was not considered for further testing. Butdotitanium, which belongs to the class of bis(β-dicetonato) metal complexes, also attracted the interest of researchers due to the cis-position of its ethoxide ligands. An important aspect of this compound is its planar aromatic system (Figure 29). It has high reactivity and has been tested extensively. The most recent ones indicate that the compound binds to DNA by intercalation, and that the amount of DNA binding is related to tumour inhibitory potential. Leukocytopenia and thrombocytopenia have not been observed, and the maximum recommended dose is 230 mg/m^2^. At higher doses, arrhythmias occur. Its use is limited by its hepatotoxicity [150].

## 7. Ruthenium Complexes

There is growing interest in the development of new metal compounds that can serve as antineoplastic drugs, with lower toxicity, fewer resistance problems and greater or equal effectiveness to platinum compounds. One of the most promising metals for the development of anti-tumour drugs is ruthenium. Ruthenium is a transition metal in group 8 of the periodic table, the ion has octahedral coordination geometries and the two main oxidation states of the metal are (II) and (III). The thermodynamic and kinetic stability of Ru(II) compounds is greater than that of Ru(III) compounds. Ruthenium also forms compounds with higher oxidation states such as (IV), but they are generally not used for therapeutic purposes due to their instability [151].

In recent years, different organometallic ruthenium complexes have been found to exhibit antitumour activity. The mechanisms of action of Ru(II) and (III) complexes are varied and depend on the nature of the ligands they possess and their target. The solubility of these compounds can be improved by modifying the ligand structures or by encapsulating the ruthenium compounds in nanomaterials.

### 7.1. Ru(III) Complexes

Some ruthenium complexes have been shown to possess unique properties in terms of antitumour activity and selectivity. As with Pt(IV) complexes, these Ru(III) derivatives are therefore prodrugs that require reductive activation to become cytotoxic Ru(II) species. The relatively inert Ru(III) species are transported via their affinity for transferrin and delivered to tumours, where they are reduced to the Ru(II) state with preference over healthy tissues due to the combined effects of hypoxia and acidic environment [152].

Ruthenium complexes appear to interact with DNA by a mechanism similar to that of platinum. They are thought to bind to the N7 of guanine which is complemented by arene intercalation bonds as well as hydrogen bonds between the chelate ligand and the C6O of guanine [153,154]. In general, the mechanism of ruthenium complexes is still poorly understood. In some cases, a significant ability to bind to DNA has been described. In addition, if such complexes have weak ligands such as Cl- and hydrophobic ligands, two forms of binding can be expected. On the one hand, hydrolysis of the labile ligand (Cl-) generates the corresponding aquo-complexes that will allow it to covalently bind to the N7 guanine, similar to cisplatin. On the other hand, hydrophobic ligands may have a tendency to interact with the hydrophobic part of the DNA, intercalating between its strands [155].

**Figure 30:**
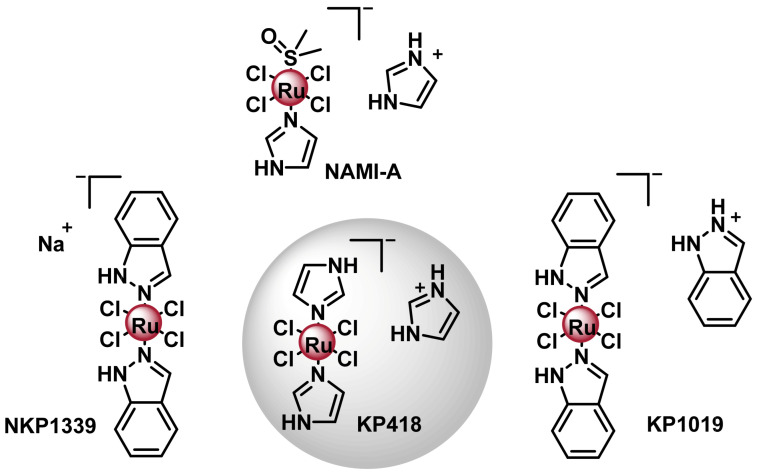
Structure of some anticancer Ru(III) complexes.

In 1986, Keppker published the synthesis of an anionic Ru(III) complex called KP418, which showed activity against the P388 leukaemia cell line and the B16 melanoma cell line implanted in BDF1 mice [156]. KP418 was the starting point for other complexes such as NAMI-A, KP1019 and NKP1339 that share the four chloride ligands found in the equatorial plane, and the axial ligands and counterions are modified [157] (Figure 30).

#### 7.1.1. NAMI- A

Imidazolium trans-imidazoledimethylsulfoxidetetrachlororuthenate, a Ru(III) compound called NAMI-A, was the first ruthenium compound tested in humans (1999) in a phase 1 clinical trial for the treatment of small cell lung cancer and reached phase II clinical trials in 2008, an important starting point in the development of ruthenium anticancer drugs. NAMI-A, under physiological conditions, is rapidly reduced to the dianionic Ru(II) species by ascorbic acid or glutathione (GSH), which are found within cells [158,159]. Bergamo et al. demonstrated that DNA is not its main target and that only a small amount of the complex is able to enter the cell interior, which is in agreement with its low cytotoxicity [160]. In 2011 it was discarded due to its reduced efficacy.

#### 7.1.2. KP-1019/NKP1339

The KP1019 complex, or [indazolium trans-[tetrachlorobis(1H-indazole)ruthenate(III)], is a Ru(III) complex developed by Keppler’s group in 1992, containing two indazole hetero-cycles coordinated to the metal centre via the nitrogen atoms at position 2 [161].

This ruthenium complex was the second agent to be tested in humans and passed phase I clinical trials. KP1019 has been shown to exhibit direct cytotoxic activity, promoting apoptosis in numerous cancer cell lines as well as in different tumour models, and is particularly active in the treatment of colorectal cancers. This compound completed a preliminary phase-I study in 2006 with promising results but failed to make progress due to a major solubility issue [162].

The solution to this solubility problem was the synthesis of the sodium salt, NKP1339. This new complex is 30 times more soluble than its predecessor, thanks to the simple exchange of the indole cation for the sodium cation. It successfully completed phase I clinical trials in 2016. NKP1339 is currently in phase I/II clinical trials [163]. NKP1339 is able to partially control neuroendocrine tumours in selected patients and is partially active against colon cancer [164].

### 7.2. Ruthenium(II) Complexes

Ru(II) complexes have very diverse ligands and structures and consequently have different mechanisms of action and different therapeutic targets. Recent advances in photodynamic therapy (PDT) and nanomaterials have made it possible to prepare bioactive Ru(II) complexes with photophysical properties, improving their efficacy and selectivity in the body [165].

#### 7.2.1. Ruthenium(II) Complexes with Arene Ligands

**Figure 31:**
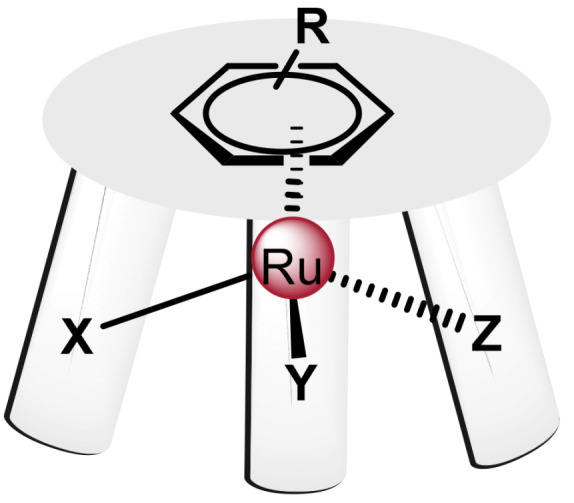
Typical generic structure of a piano stool compound with three monodentate ligands.

Ru(II) arene complexes are organometallic complexes with pseudo-octahedral geometry, are also known as “piano stool” and have the general formula [(6-arene)Ru(X)(Y)(Z)], (Figure 31). The arene forms the seat of the stool, while the remaining three positions would be occupied by the other ligands, which would co-respond to the legs of the stool, which can be a tridentate ligand, a bidentate ligand and a monodentate ligand or three monodentate ligands [166].

There is great interest in the synthesis of this type of compounds, due to the possibility of being able to vary each of the components. The arene rings can be of various natures, including benzene, biphenyl or dihydroanthracene, among others. This arene group determines the electron distribution of the Ru(II) complex, influencing its stability, as well as facilitating the entry of these agents into cells due to the hydrophobicity of these rings. The ease of hydrolysis of the Ru-Z bond is affected by the pH and the concentration of the Z-ligand in the medium. The water solubility and volume of the chelating ligand and leaving group can also affect the efficacy of these agents [151].

**Figure 32:**
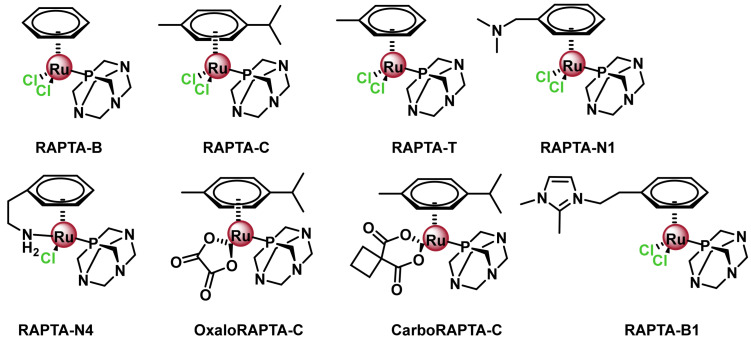
RAPTA complexes.

The most studied Ru(II) arene complexes, as new anti-cancer complexes, are due to Dyson and Sadler with the development of the RAPTA-type complexes of general formula [(Z6-arene) Ru(X)(Y)(PTA)], [167,168,169,170], Figure 30 where X and Y are normally chlorides, and PTA is the monodentate phosphate ligand, 1,3,5-triaza-7-phosphaadamantane (1,3,5-triaza-7-phosphatidecyl-[3. 3.1.1]decane), which has good aqueous solubility and is preferentially protonated at low pH. Its pharmacological properties can be easily modulated by ligand modification (Figure 32).

**Figure 33:**
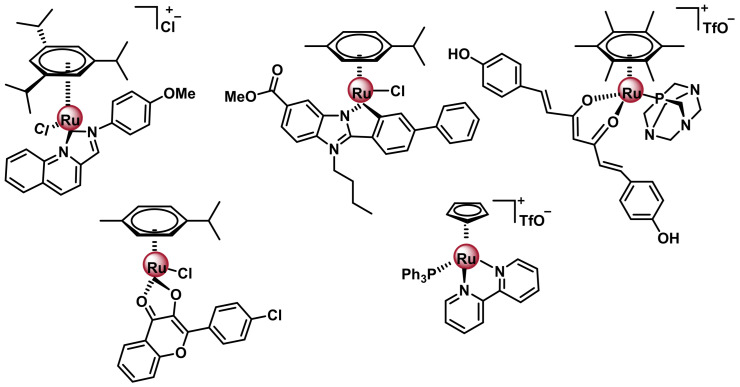
RAPTA family of anticancer agents.

RAPTA complexes have been reported to exhibit a spectrum of activity similar to that of NAMI-A, despite their differences in oxidation state, ligands, charge and geometry [171]. Phosphoadamantane-type RAPTA complexes have been tested in clinical trials because they exhibit remarkable anticancer activity and can be tuned by careful selection of the ligand sphere [172,173,174,175,176]. Following the good properties of the RAPTA family complexes, new Ru(II) complexes with three monodentate ligands have been described with modifications both in the arene ligand by introducing substituents such as electrodentate groups, electroacceptor groups or aromatic rings and in the chloride ligands by substituting them with other monodentate ligands [177] (Figure 33).

#### 7.2.2. Octahedral Ruthenium(II) Complexes with Polypyridine Ligands

Ru(II) complexes with polypyridine ligands in general possess excellent reactivity and a great ability to act as imaging agents and redox chemistry, which makes them potential drugs for cancer diagnosis and therapy. These agents can act reversibly as probes or inhibitors of important biological molecules such as DNA, RNA or proteins [178].

**Figure 34:**
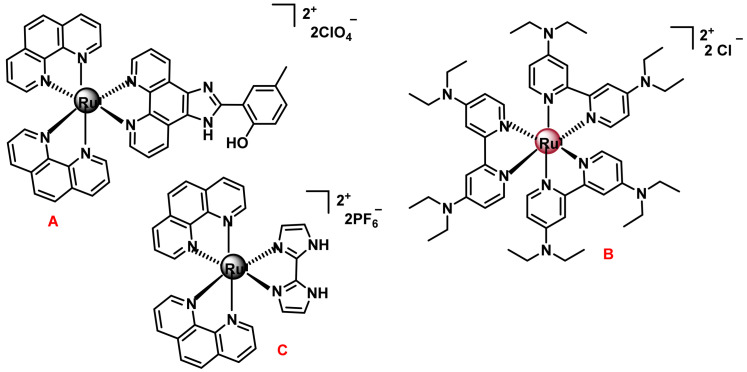
Examples of Ru(II) polypyridyl complexes with antitumor activity.

The most characteristic Ru(II) complex is the compound [Ru(bpy)3]^2+^(bpy = 2,2’-bipyridine)] which presents a series of interesting physicochemical characteristics since it is kinetically stable in the dark and presents an absorption band in the visible region; if excited, it emits in the visible region of the spectrum. These optical properties allow the tracking and localisation of the complexes inside the cell by confocal microscopy, giving them ideal characteristics for their biological study and for their application as a diagnostic agent. for their application as a diagnostic agent. These optical properties, together with the versatility of modifying the ligands, either by changing the atoms through which they coordinate, by introducing electro-doping or electro-acceptor groups, or by conjugation to biomolecules, make them good candidates for cancer therapy [179]. Three examples of polypyridyl Ru(II) complexes with anti-tumour activity are shown in Figure 34.

Complex A has suitable characteristics for study by confocal microscopy, and accumulates preferentially in the endoplasmic reticulum, being its therapeutic target. The optical properties of complex B show that it accumulates preferentially in cell membranes, but not inside the nucleus, ruling out this being its therapeutic target, although its mechanism of action has not yet been determined.

**Figure 35:**
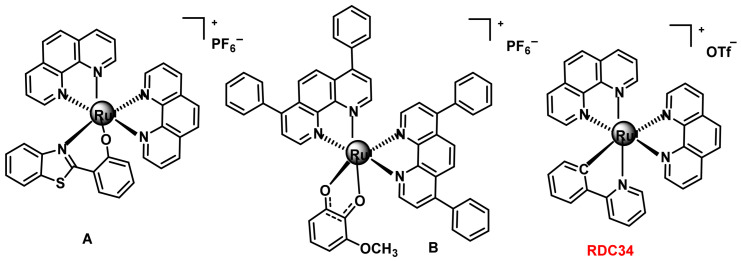
(**A**) Octahedral Ru(II) complexes containing O^N^ ligands, (**B**) coordinated O^O^ and RDC34 with a cyclometalated ligand.

The mechanism of action of the C-complex is the induction of apoptosis in tumour cells through disruption of the membrane potential. There are also octahedral Ru(II) complexes containing two chelated N^N^ ligands and a ligand coordinated through other heteroatoms, such as O^N^, O^O^ or C^N^, (Figure 35).

Complex A containing two 1,10-phenanthroline ligands and a 2-(2-hydroxyphenyl) benzothiazole ligand [180]. This complex is active against the lung tumour line A459. The therapeutic target of this complex is the mitochondria. The B-complex with a catechol-based ligand containing a methoxy electrode group has a dual action, since both DNA and mitochondria are its main therapeutic targets [181]. The octahedral Ru(II) complex with a cyclometallated ligand, such as the RDC34 complex which contains two 1,10-phenanthroline ligands and a cyclometallated 2-phenylpyridine ligand. The RDC34 complex is cytotoxic in the glioblastoma cancer cell line A172, while it is less active in healthy glia and neuronal cells obtained from mouse cerebellum [182].

#### 7.2.3. Polypyridyl Ru(II) Complexes as Photosensitisers (PS) in Photodynamic Therapy (PDT)

Polypyridyl-type Ru(II) complexes, whether coordination complexes or organometallic complexes, have properties that make them suitable for use in PDT applications. Photodynamic therapy is a therapeutic technique approved for the treatment of cancer that aims to exert its action in a localised manner while reducing side effects. The basis of this therapy is based on the use of a molecule capable of being activated by light, called photosensitiser visible light and molecular oxygen [183]. Photodynamic therapy consists of two steps: first, the PS is administered so that it accumulates in the cancerous tissues and, in the second step, it is exposed to light. These two independent steps reduce side effects because the photosensitiser, which is non-toxic in the dark, is only activated when irradiated with light that is concentrated on the specific area where the tumour is located, destroying it. This is a way of reducing side effects as the PS only acts on the irradiated area and not on healthy tissue [184].

Some polypyridyl-type Ru(II) complexes may act as agents for teragnosis, as they can exert a therapeutic function, act as a photosensitiser of PDT, and as a bioimaging agent for diagnosis [185].

**Figure 36:**
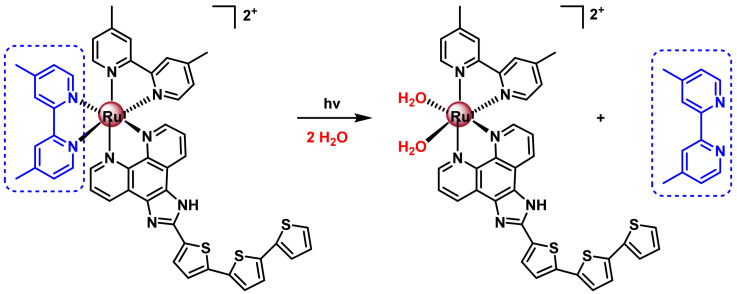
Molecular structure of the PS complex of Ru(II) TLD4133. Activation of the TLD-1433 complex.

The TLD-1433 complex, described by S. McFarland’s research group, 2020, is the first Ru(II)-based photosensitiser for photodynamic therapy to enter human clinical trials for the treatment of muscle-invasive bladder cancer [186]. This complex has two 4,4’-dimethyl-2,2’-bipyridine ligands and an imidazole [4,5-f]-1,10-phenanthroline ligand substituted with three thiophene rings. The activation of this complex consists of the loss of a ligand, photoinduced by visible light in water (Figure 36), activating the complex by forming covalent bonds with guanosine monophosphate, through the substitution of labile Ru-OH_2_.

**Figure 37:**
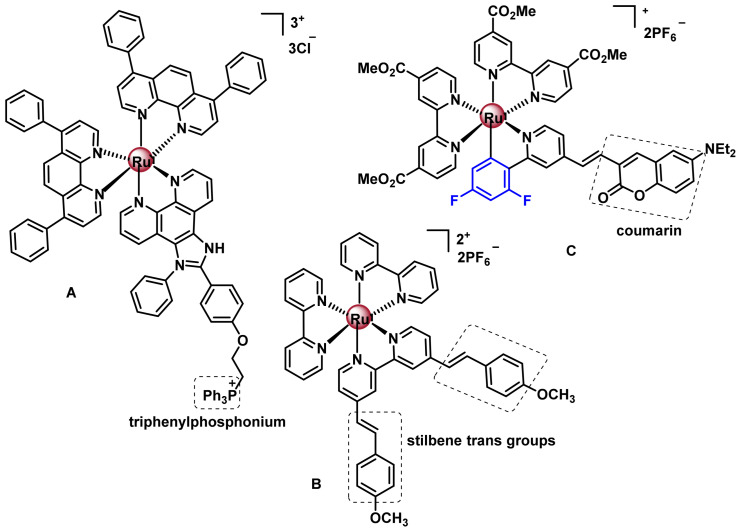
Ru(II) polypyridyl PS complexes for PDT. (**A**) Ru(II) polypyridyl complex containing a triphenylphosphonium substituent. (**B**) 2,2’-bipyridine type complex with trans-stilbene groups in the 4’ and 4‘ positions of the pyridines. (**C**) Cyclometalated Ru(II) complex for PDT.

From the study with TLD1433, new Ru(II) complexes were developed for use in PDT, (Figure 37). Chao’s research group developed the polypyridyl Ru(II) complex containing a triphenylphosphonium (A) substituent to target the compound to mitochondria [187]. It is active in two-photon PDT.

Complex B was described by Gasser’s research group. This complex has 2,2’-bipyridine ligands with trans-stilbene groups at the 4 and 4‘-positions of the pyridines. These stilbene derivatives are effective for two-photon irradiation. In addition, electrocatalytic substituents, such as the methoxy group, have been included in order to improve the electronic properties of the complex. This complex is able to penetrate into the cell interior through a process of endocytosis and accumulates mainly in the cytoplasm [188].

The Ru(II) C complex, with a cyclometallated ligand, possesses a coumarin fragment and shows absorption bands in the 400–700 nm range. This PS remains active under hypoxic conditions [189].

#### 7.2.4. Ru(II) Complexes for Photoactivated Chemotherapy (PACT)

Photoactivated chemotherapy is another alternative to improve the selectivity and reduce the side effects of Ru(II) chemotherapeutic agents. PACT is based on the use of a prodrug that is inactive in the dark and, when irradiated with light, is activated and transformed into the drug that exerts the cytotoxic effect [190]. The activation mechanism is independent of oxygen concentration, which makes it possible to maintain the activity of the complexes in hypoxic conditions, which is one of the major drawbacks of PDT. The two possibilities for photoactivation are oxidation or reduction of the metal centre [191].

**Figure 38:**
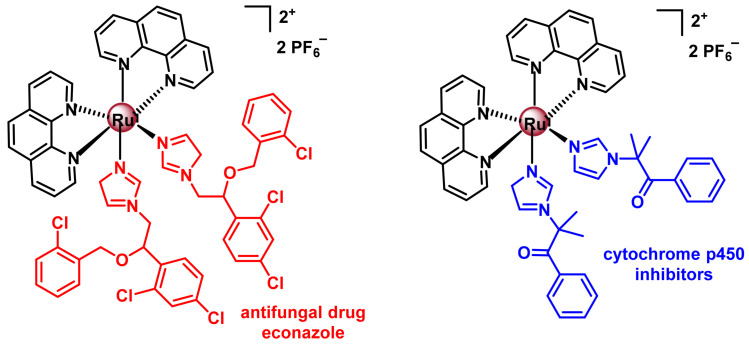
Ru(II) complexes for PDT containing monodentate ligands enzyme inhibitors. Left complex synthesized by Renfrew and right by Glazer.

Polypyridyl Ru(II) complexes for use in PACT must contain bioactive ligands that, when coordinated to the metal complex, produce no effect but, upon photoliberation, reach their target and perform their cytotoxic effect, (Figure 38). Renfrew et al. published a polypyridyl Ru(II) complex containing two ligands of the antifungal drug econazole [192]. This complex is luminescent, allowing it to be localised inside the cell by confocal microscopy. The cytotoxic activity of this complex has been studied in different tumour cell lines of breast MCF-7, prostate LNCaP and PC-3 and colon DLD-1, since econazole is able to induce apoptosis and reduce tumour size in in vivo models with mice with these types of cancer.

Glazer et al., 2016, prepared the compound containing two ligands that act as co-enzyme inhibitors of cytochrome p450, which is an enzyme that plays an essential role in catalysing metabolic biosynthesis reactions involving steroids, retinoic acid or vitamin D and is also responsible for drug metabolism. This enzyme is particularly overexpressed in tumours and plays a direct role in cancer initiation, progression and resistance [193].

**Figure 39:**
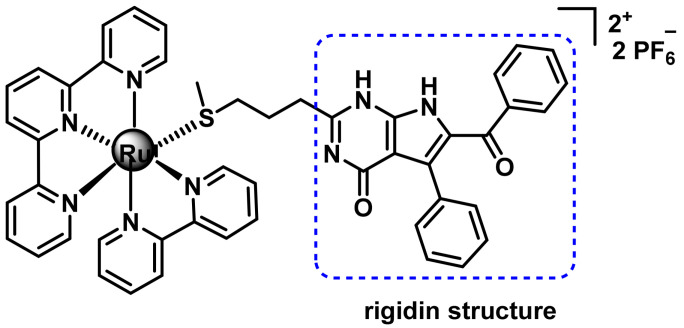
Ru(II) complex with a monodentate ligand coordinated through a sulfur atom.

The development of a complex with a terpyridine ligand, a 2,2’-bipyridine ligand and a monodentate ligand containing the rigidin structure, which has been modified to coordinate to the ruthenium atom via the sulphur atom of a thioether group, has been synthesised and studied by Bonnet’s group, 2019 [194], (Figure 39).

Rigidin is an alkaloid used as a drug, whose therapeutic target is the inhibition of tubulin polymerisation, which prevents cells from generating microtubules and forming their cytoskeleton, impeding their development. The complex is not able to inhibit tubulin polymerisation, but when irradiated with green light it photodissociates the monodentate ligand and this acts to inhibit polymerisation [194].

Ruthenium complexes are promising not only for their anticancer potential, but also for their antimicrobial activity, which is based on common underlying mechanisms. These complexes often act through DNA binding, generation of reactive oxygen species and disruption of cellular processes, which are effective against both cancer cells and microbial pathogens [195,196]. In a recent study, the researchers synthesized and characterized a novel bioconjugate consisting of buforin II, an antimicrobial peptide, and buforin II–[Ru(bpy)_3_]^2+^ bioconjugate. The bioconjugate showed submicromolar activity against the multidrug-resistant strains *Escherichia coli* AR 0114 and *Acinetobacter baumannii* Naval-17, indicating a strong synergistic effect between buforin II and the ruthenium complex. Mechanistic studies showed that bioconjugate 1 significantly increased the rate of DNA damage in target cells compared to [Ru(bpy)_3_]^2+^ alone [197]. The combination of Ru complexes with AMPs makes use of these synergistic mechanisms and offers a versatile approach to therapy.

## 8. Gold Complexes

In the 20th century, chrysotherapy (the use of gold-based drugs for the treatment of diseases) was accepted as part of modern medicine. Gold complexes are of great importance in modern medicine. Although gold metal complexes can adopt oxidation states from −1 to +5, gold is present in two main oxidation states: Au(I) and Au(III) [198] and several complexes with high anti-cancer potential have been developed. The oxidation state of the gold atom influences the geometry and chemistry of the compound, as well as the ligands that can be coordinated to the metal centre and thus the final anticancer properties [199].

### 8.1. Gold(I) Complexes

They have a d^10^ electronic configuration, with linear geometries with two ligands coordinated to the gold atom. Au(I) is a soft metal centre, so it tends to form stable complexes with soft donor atoms such as sulphur, selenium or phosphorus [200].

**Figure 40:**
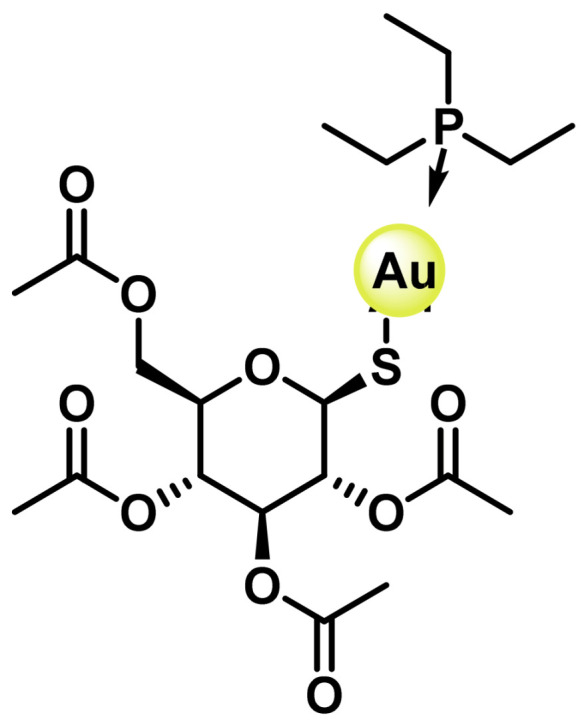
Auranofin [(tetra-O-acetyl-β-D-glucopyranosyl)-thio] (triethylphosphine)-Au(I).

Auranofin (2,3,4,6-tetra-6-acetyl-1-thio-β-D-glucopyranosate-S-triphenylphosphine Au(I) has an Au(I) metal centre, with two ligands coordinated to it: a triethylphosphine molecule and a thioglucose group, (Figure 40). The phosphine gives it a lipophilic character, so it is more soluble than other gold complexes, allowing it to be administered orally rather than intravenously [201].

**Figure 41:**
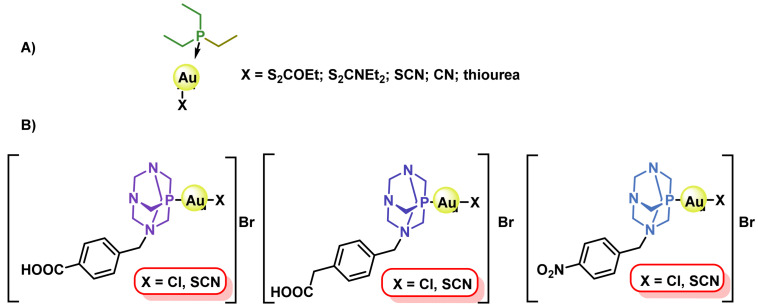
(**A**) Linear Au(I) complexes similar to auranofin. (**B**) Au(I) complexes with phosphines derived from 1,3,5-triaza-7-phosphaadamantane.

It began to be used as an antitumour agent when it was discovered that patients with rheumatoid arthritis who had been treated with auranofin were less prone to developing tumours [202]. This fact, together with the discovery of the antitumour properties of cisplatin and its analogues, encouraged researchers to evaluate the effect of auranofin on various tumour lines [203,204,205,206,207,208], finding that it has less nephrotoxicity than cisplatin and that it could be used at a dose three times higher to be lethal and also has fewer side effects than other metals used in cancer treatment, and this was the starting point for the synthesis of a variety of gold complexes for the treatment of cancer [198,209], (Figure 41).

Au(I) complexes are thermodynamically more stable and less susceptible to reduction to metallic gold than Au(III) complexes. Au(I) complexes generally have a linear geometry. The anti-cancer potential of Au(I) complexes has been the subject of much research in recent years [210].

Au(I) complexes have a lower affinity for DNA than cisplatin and its analogues under physiological conditions [211], because Au(I) is a soft acid, but is particularly attracted to sulphur and selenium atoms, and less attracted to nitrogen and oxygen than platinum derivatives [212]. These complexes interact with the thiol groups of proteins and peptides, mainly those containing sulphur and/or selenium such as selenoproteins. The difference between Au(I) complexes and oxaliplatin is the mode of activation; this class of gold complexes does not usually undergo activation processes; however, the ligands can be exchanged for biomolecules once inside the cell [209].

Auranofin was approved by the FDA in 1985, and is currently in clinical trials for leukaemia, lung, breast and ovarian cancer. Both in vitro and in vivo models have demonstrated its antitumour activity, which is based on its ability to inhibit thioredoxin reductase (TrxR), a flavoenzyme that together with thioredoxin (Trx) forms one of the major antioxidant defence systems required for cell survival, and is involved in redox homeostasis, transcription, regulation of cell proliferation and replication. Overexpression of TrxR is associated with aggressive tumour progression and poor survival in patients with breast, ovarian and lung cancer [213].

The Au(I) complexes that have been synthesised and evaluated over the last decade share with auranofin the ability to inhibit TrxR, and great efforts have been made to improve the complex-enzyme interaction and thus increase the effectiveness of the therapy [214,215]. In addition, several derivatives have been successfully tested on cisplatin-resistant cell lines, pointing to a possible use of Au(I) complexes in tumours resistant to traditional therapeutic strategies [216,217].

**Figure 42:**
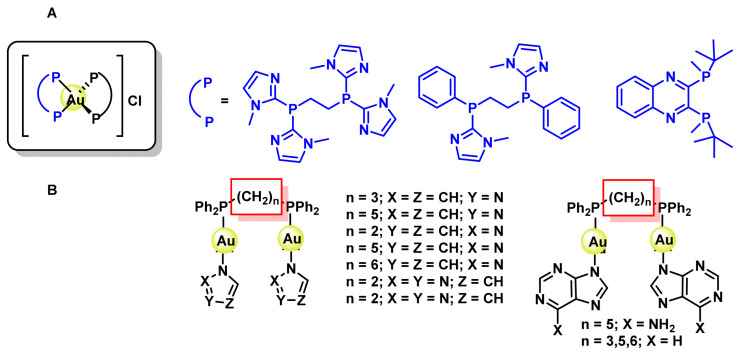
(**A**) Structures of mononuclear and (**B**) dinuclear complexes with diphosphines.

Complexes with bi-dentate phosphines have also been prepared and evaluated for their activity, providing excellent in vitro results against different colon cancer cell lines [218] and bidentate phosphines as ligands bridging two Au(I) centres coordinated to molecules such as imidazoles, pyrazoles, triazoles and pyrroles and whose activity depends on the length of the diphosphine chain [219] (Figure 42).

**Figure 43:**
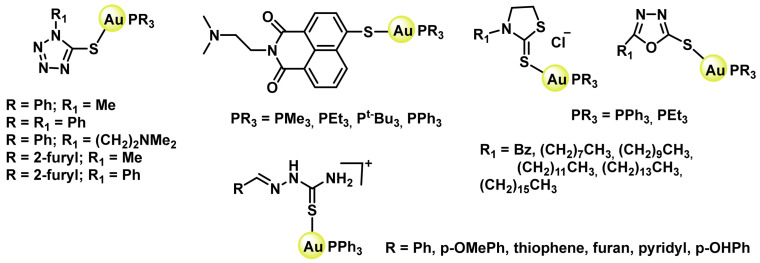
Au(I) complexes with donor sulfur ligands.

S forms stable complexes with Au(I), coordinating different ligands and generating a wide variety of complexes with anticancer potential; among these ligands are naphthalimide tetrazoles [220,221], heterocyclic compounds and aryl-thiosemicarbazones [222], (Figure 43).

**Figure 44:**
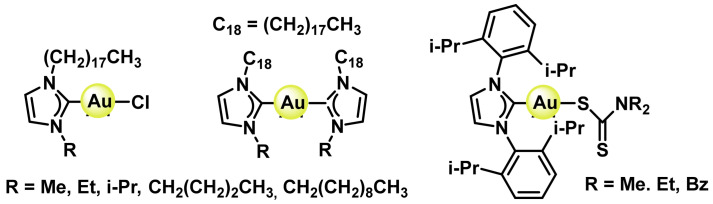
Au(I) complexes with carbenes derived from 1,3-disubstituted imidazole.

A wide variety of Au(I) complexes with NHCs (N-heterocyclic carbenes) ligands have been synthesised, most of them being 1,3-disubstituted imidazole derivatives, (Figure 44). These metal derivatives yielded promising cytotoxic results [215,223,224].

**Figure 45:**
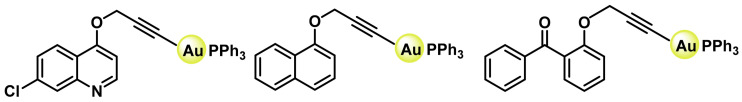
First Au(I) derivatives with alkynyl ligands for the treatment of colon cancer.

Alkynyl derivatives of Au(I) have also been described, the alkynyl ligands act as strong σ- and π-ligands giving rise to complexes with high stability, in fact the gold-carbon bond in gold complexes with alkynes is one of the strongest gold-ligand bonds known. These alkynyl Au(I) derivatives were first used as anti-tumour agents against colon cancer in 2009 [225], (Figure 45).

**Figure 46:**
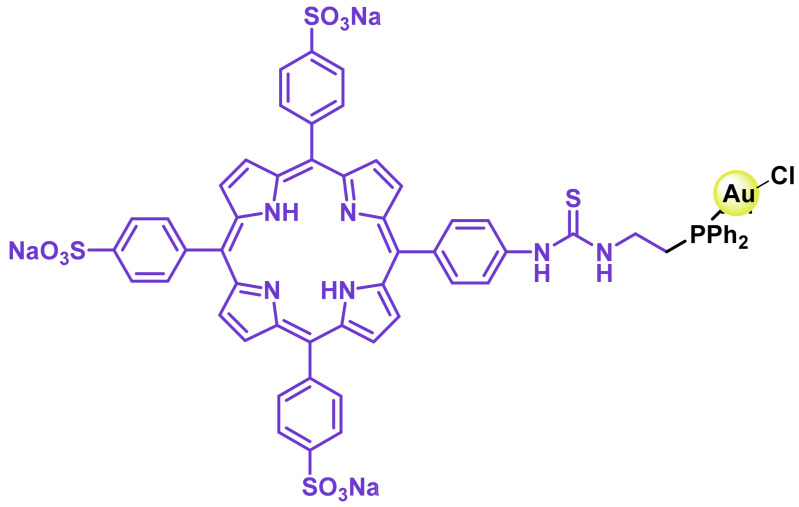
Au(I) complex with a porphyrin-derived phosphine ligand.

Tasan et al. prepare a complex in which phosphine can be used as a linker to bind other molecules that give the gold complexes a desired characteristic, (Figure 46), thus adding the diagnostic capability of porphyrin to the final gold complex [226].

### 8.2. Gold(III) Complexes

Au(III) complexes could present significant advantages in the field of chemotherapy, since it is isoelectronic with cisplatin and both possess a d8 electronic con-figuration, giving a square planar geometry. It was thought that Au(III) complexes might possess a similar mechanism of action to cisplatin, as well as a comparable therapeutic effect [209,227].

Although a priori less stable than Au(I) complexes, an appropriate choice of coordination ligands has made it possible to synthesise numerous Au(III) derivatives with interesting anticancer potential, as can be seen in extensive literature reviews carried out in recent years [227,228,229,230,231,232,233,234]. Given their similarities to cisplatin, it could be thought that the use of these derivatives could also give rise to serious side effects due to a hypothetical ability to interact with DNA. Although some cases have been described in which direct interaction with this macromolecule seems to be one of its mechanisms of action 178–180, other different molecular targets of great interest in therapy have also been identified [235,236,237].

For example, some Au(III) derivatives have the ability to induce a decrease in cyclin B1 expression levels, which induces a cessation of cell cycle progression in the G2/M181 phase, or the affinity of other complexes in this class to inhibit the enzyme poly ADP ribose polymerase 1 (PARP-1). These studies show that, despite the shared characteristics of cisplatin and Au(III) complexes, the latter have greater anti-tumour potential in terms of the molecular targets with which they can interact, so they have an interesting and promising future in chemotherapy [238,239].

**Figure 47:**
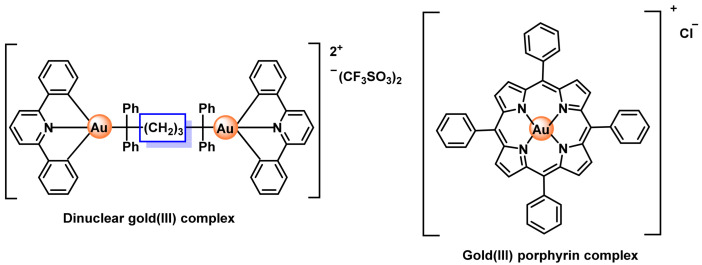
Dinuclear Au(III) complex (left image) and porphyrin Au(III) complex (right image) designed by Che et al. [240].

In the dinuclear Au(III) complex (Figure 47), which has been shown to have selective cytotoxicity against tumour lines, the length of the alkyl chain separating the phosphine groups attached directly to the gold atoms is very important for this activity [240].

The in vivo study showed 77% inhibition of tumour growth at a dose of 10 mg/kg intraperitoneally twice weekly. In addition, treated animals showed no adverse side effects such as weight loss and diarrhoea at the effective dose. The complex also showed low genotoxicity, as determined by analysing the mutagenicity of polychromatic erythrocytes from treated mice. Au(III) porphyrin was developed by the same group and they found that was able to induce apoptosis in vitro in cisplatin-resistant ovarian cancer, giving also good results in vivo [241].

**Figure 48:**
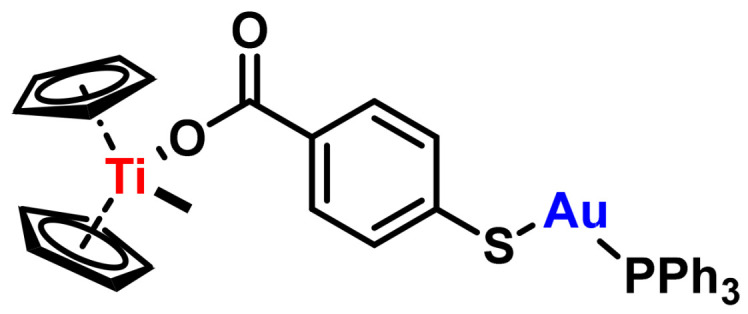
Ti-Au heterometallic complex.

Structure-activity relationship analysis suggested that complexes that could overcome cisplatin resistance generally possess a net cationic charge and can be stabilised by porphyrin acting as electron donors or 2,6-diphenylpyridine. In the search for metal complexes that exceed the efficacy of cisplatin, the Contel team has successfully synthesized a novel titanocene complex with Au(I) fragments in its structure (Figure 48).

This heterometallic complex was able to reduce the original tumour size by up to 67% and produced very few toxic effects. Unfortunately, the pharmacokinetic study revealed accumulation of this complex in the kidney and liver [242].

## 9. Drug Delivery Systems and Nanotechnology

In recent years, in the fight against different types of cancer, nanostructured materials have been used for the controlled release of cytostatic drugs directly in the affected area, macromolecular systems such as cyclodextrins, liposomes, lipid nanocapsules, adducts of metal complexes with proteins, polynuclear complexes, ceramic-type materials based on calcium phosphate, carbon nanotubes or mesoporous silicas.

The application of nanoparticles in cancer treatment is known as nano-oncology. Nanoparticles can decrease off-target drug distribution and associated side effects [243,244,245]. The advantages over direct drug delivery are:

(i). improved biodistribution,
(ii). improved bioavailability and solubility of drugs,
(iii). decreased toxicity,
(iv). prevention of multi-drug resistance,
(v). increased drug stability, prevent degradation of the encapsulated cargo,
(vi). allow encapsulation of a larger amount of drugs, [246]
(vii). cross barriers (such as the blood-brain barrier) due to their small size, and
(viii). to carry out therapies directed at specific targets, which translates into the use of lower doses of drugs and therefore lower toxicity by facilitating their accumulation in tumours through the effect of improved permeability and retention [247].

The use of cisplatin-loaded nanoparticles with iron metal ions have shown increased antitumour efficacy and reduced side effects compared to free cisplatin in preclinical studies [248]. Ruthenium complexes encapsulated in liposomes or polymeric nanoparticles have shown enhanced anti-cancer and tumour-targeting activity in vitro and in vivo [249,250].

Another way to functionalise nanocarriers is with targeted ligands, such as antibodies, peptides or small molecules, to specifically recognise and bind to overexpressed receptors on tumour cells, minimising damage to healthy tissue and improving overall therapeutic outcomes. For example, folic acid-conjugated gold nanoparticles loaded with a Au(III) complex have demonstrated enhanced tumour uptake and cytotoxicity in vitro and in vivo compared to free Au(III) complex [251].

Stimuli-responsive materials have also been used in drug delivery systems to enable the controlled and targeted release of metallodrugs in response to specific physiological or pathological stimuli, such as pH, temperature, enzymes or redox conditions. One example is the development of pH-sensitive polymeric nanoparticles for targeted delivery of cisplatin. Stimuli-responsive drug delivery systems can help overcome drug resistance and limited penetration, further enhancing the therapeutic potential of metallopharmaceuticals [252].

Carbon-based nanomaterials, gold nanoparticles, coordination polymers, organometallic structures, micelle polymers, etc. are used as platinum (IV) nanodelivery against cancer [253,254,255,256,257]. These nanoparticles are generally absorbed by cancer cells because of their higher permeability and higher drug retention effect [258,259,260].

The Ru(II) complexes encapsulated in nanosystems, once administered into the bloodstream, improve their directionality and preferentially accumulate in solid tumours, due to the effect of permeation and retention. The main Ru(II) nanosystems are nanoparticles based on selenium, gold, silica or carbon nanotubes.

With the use of nanosystems, the vectorisation of drugs is being achieved, resulting in improved effectiveness and reduced side effects.

The challenges posed by the use of nanocarriers are: (i) the need to optimise their physicochemical properties, (ii) to control the kinetics of drug release, (iii) to ensure biocompatibility and safety [261].

## 10. Conclusions

The use of metallodrugs continues to be a field of great interest and many reports are being published on this topic. Current data on metallodrugs support the idea that they may function as chemotherapeutic agents, due to their potential to inhibit signalling pathways in multiple aspects of cancer progression, including tumour growth, angiogenesis and metastasis.

The limitations of metallodrugs in terms of side effects, low solubility and low bioavailability in the human body, due to their low hydrolytic stability, are very difficult to address from a monodisciplinary point of view. However, these problems associated with metallodrugs can be overcome by using mixed metallodrug and nanotechnology approaches, such as encapsulation of metal-based drugs in different nanostructured materials. For example, the use of liposomes, lipid nanocapsules, human proteins, ceramic materials, carbon nanotubes and metal nanoparticles or metal oxides with anticancer metallodrugs should be of great interest. In addition, metallized host defense peptides have great potential for use in cancer therapy as they can enhance bioactivity and extend their mechanistic capabilities. This offers a promising opportunity to produce more effective anti-cancer therapeutics. Therefore, by combining nanomaterials and metallodrugs to obtain more potent and reliable formulations, we can predict that a bright future still awaits meta-les-based drugs in cancer chemotherapy.

A significant number of natural products have significant potential to protect against cisplatin-induced toxicity in multiple organs, including the liver, kidneys, cardiovascular, haematopoietic, reproductive and nervous systems. The application of natural prodrug-based formulations of cisplatin is a new therapeutic strategy to combat human cancer. Further research on current metallodrugs or even the development of new metallodrugs is needed.

## Data Availability

Data sharing is not applicable.
